# Extending the Proteomic Characterization of Candida albicans Exposed to Stress and Apoptotic Inducers through Data-Independent Acquisition Mass Spectrometry

**DOI:** 10.1128/mSystems.00946-21

**Published:** 2021-10-05

**Authors:** Ahinara Amador-García, Inés Zapico, Ana Borrajo, Johan Malmström, Lucía Monteoliva, Concha Gil

**Affiliations:** a Department of Microbiology and Parasitology, Faculty of Pharmacy, Complutense University of Madrid (UCM), Madrid, Spain; b Proteomics Unit, Complutense University of Madrid, Madrid, Spain; c Division of Infection Medicine, Department of Clinical Sciences, Lund University, Lund, Sweden; d Ramon y Cajal Health Research Institute (IRYCIS), Madrid, Spain; NYU School of Medicine

**Keywords:** *C. albicans*, oxidative stress, acetic acid, proteomics, data-independent acquisition, selected reaction monitoring, apoptosis, proteasome

## Abstract

Candida albicans is a commensal fungus that causes systemic infections in immunosuppressed patients. In order to deal with the changing environment during commensalism or infection, C. albicans must reprogram its proteome. Characterizing the stress-induced changes in the proteome that C. albicans uses to survive should be very useful in the development of new antifungal drugs. We studied the C. albicans global proteome after exposure to hydrogen peroxide (H_2_O_2_) and acetic acid (AA), using a data-independent acquisition mass spectrometry (DIA-MS) strategy. More than 2,000 C. albicans proteins were quantified using an ion library previously constructed using data-dependent acquisition mass spectrometry (DDA-MS). C. albicans responded to treatment with H_2_O_2_ with an increase in the abundance of many proteins involved in the oxidative stress response, protein folding, and proteasome-dependent catabolism, which led to increased proteasome activity. The data revealed a previously unknown key role for Prn1, a protein similar to pirins, in the oxidative stress response. Treatment with AA resulted in a general decrease in the abundance of proteins involved in amino acid biosynthesis, protein folding, and rRNA processing. Almost all proteasome proteins declined, as did proteasome activity. Apoptosis was observed after treatment with H_2_O_2_ but not AA. A targeted proteomic study of 32 proteins related to apoptosis in yeast supported the results obtained by DIA-MS and allowed the creation of an efficient method to quantify relevant proteins after treatment with stressors (H_2_O_2_, AA, and amphotericin B). This approach also uncovered a main role for Oye32, an oxidoreductase, suggesting this protein as a possible apoptotic marker common to many stressors.

**IMPORTANCE** Fungal infections are a worldwide health problem, especially in immunocompromised patients and patients with chronic disorders. Invasive candidiasis, caused mainly by C. albicans, is among the most common fungal diseases. Despite the existence of treatments to combat candidiasis, the spectrum of drugs available is limited. For the discovery of new drug targets, it is essential to know the pathogen response to different stress conditions. Our study provides a global vision of proteomic remodeling in C. albicans after exposure to different agents, such as hydrogen peroxide, acetic acid, and amphotericin B, that can cause apoptotic cell death. These results revealed the significance of many proteins related to oxidative stress response and proteasome activity, among others. Of note, the discovery of Prn1 as a key protein in the defense against oxidative stress as well the increase in the abundance of Oye32 protein when apoptotic process occurred point them out as possible drug targets.

## INTRODUCTION

Candida albicans is a common opportunistic fungus in the human microbiota that can cause severe infections in immunocompromised hosts. Candidiasis, caused mainly by C. albicans, ranges from local mucosal to systemic infections and has a noteworthy clinical impact on morbidity and mortality in intensive care unit patients (1). In spite of current antifungal therapies, it is estimated that invasive candidiasis causes 50,000 deaths worldwide every year (2). This is partially explained by the increase in the number of high-risk hosts or a late/deficient diagnosis, but it is also due to emerging resistance to antifungal drugs.

Among other pathogenicity mechanisms, C. albicans has the ability to respond and adapt to different host microenvironments (3), including a wide pH range and the antimicrobial oxidative burst originated by innate immune cells. C. albicans has developed several antioxidant mechanisms, including catalase, superoxide dismutases (SODs), and glutathione and thioredoxin systems that enzymatically detoxify O_2_ radicals such as hydrogen peroxide (4). As a member of the gut microbiota, C. albicans also has to cope with metabolites, including weak organic acids such as acetic acid, produced by other microorganisms (5). Both hydrogen peroxide and acetic acid have been described as inducers of regulated cell death in C. albicans (6–8). For this reason, the study of the response of C. albicans to these agents is a promising alternative strategy against this pathogen. Regulated cell death in C. albicans upon exposure to many agents, including antifungals and plant extracts, has been widely demonstrated (9–13). Many studies with C. albicans and Saccharomyces cerevisiae have revealed the participation of several proteins in this process, mainly from mitochondria and the Ras pathway as well as metacaspase I and its substrates (14–16). Although the terms programmed cell death and apoptosis are sometimes used interchangeably to describe regulated cell death processes, their precise meanings can be distinguished using the recommended guidelines for yeast cell death nomenclature (17). DNA fragmentation, an increase in caspase-like enzymatic activity, reactive oxygen species (ROS) accumulation, and phosphatidylserine (PS) exposure have been widely considered apoptotic markers (18, 19). However, only PS exposure is currently accepted as an apoptotic marker in yeast, and “programmed cell death” applies to cell death triggered under physiological scenarios such as aging. The term regulated cell death includes both apoptosis and programmed cell death as organized cell death processes promoted by external or internal stresses.

In order to deal with the changing environment during commensalism or infection, C. albicans must reprogram its proteome by expressing or repressing certain proteins. A better characterization of these changes in response to stress inducers is important for a deep understanding of C. albicans survival strategies, which would be very useful in the development of new antifungal therapies to combat the infection.

With this purpose, we used a data-independent acquisition (DIA) proteomic approach to identify global changes in the abundance of C. albicans proteins in response to oxidative and acetic acid stresses. This strategy for global proteomic studies is an improvement compared with the traditional data-dependent (DDA) approach, conferring better quantitative accuracy and reproducibility and enlarging the number of quantifiable peptides (20). Taking advantage of targeted proteomics as well, we used a selected reaction monitoring (SRM) method to monitor the abundance of proteins related to regulated cell death (21). This SRM method allowed the straightforward quantitation of key proteins involved in regulated cell death after exposure to the stressors hydrogen peroxide, acetic acid, and amphotericin B (AMB), an antifungal previously described as an apoptotic inducer (16, 22).

## RESULTS

### Effects of hydrogen peroxide and acetic acid on viability, physiological response, and cell death in C. albicans.

**FIG 1:**
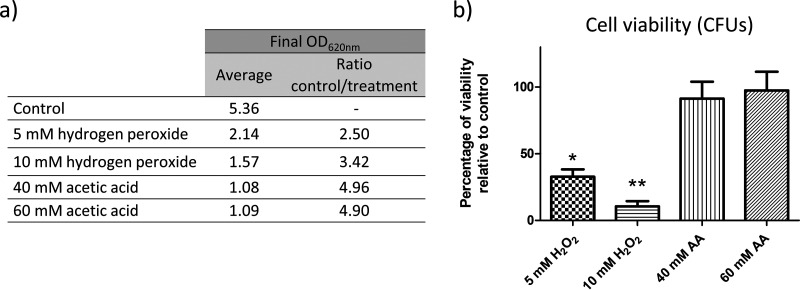
Effects on C. albicans growth after exposure to hydrogen peroxide or acetic acid. (a) Optical density reached after treatment for 200 min with the stress inducers cited. (b) Percent viability of C. albicans cells treated with the agents compared to control samples. A significant change is indicated as follows: *, *P* value < 0.05, or **, *P* value < 0.01 (paired *t* test). All results represent the averages of at least three biological replicates. AA, acetic acid.

The main goal of this study was to evaluate proteomic changes in cells exposed to different concentrations of hydrogen peroxide and acetic acid in order to reveal the main processes involved in stress responses. With the aim of calculating the damage produced by these agents to growth and cellular viability, optical density and colony formation were measured after treatment. The results show that the growth and viability of cells exposed to hydrogen peroxide were compromised in a dose-dependent manner. The final optical densities of the cell cultures after treatment with 5 mM and 10 mM hydrogen peroxide were, respectively, 2.5 and 3.4 times lower than those of the control cells, and only 35% and 13%, respectively, of the cells were viable. In contrast, acetic acid at 40 mM and 60 mM induced cell growth arrest but did not lower cell viability, as indicated by the recovery of cell growth in colonies after the treatment was stopped (Fig. 1). Despite the effects observed, the loss of cell membrane integrity as measured by propidium iodide (PI) staining was in all cases below 1%.

**FIG 2:**
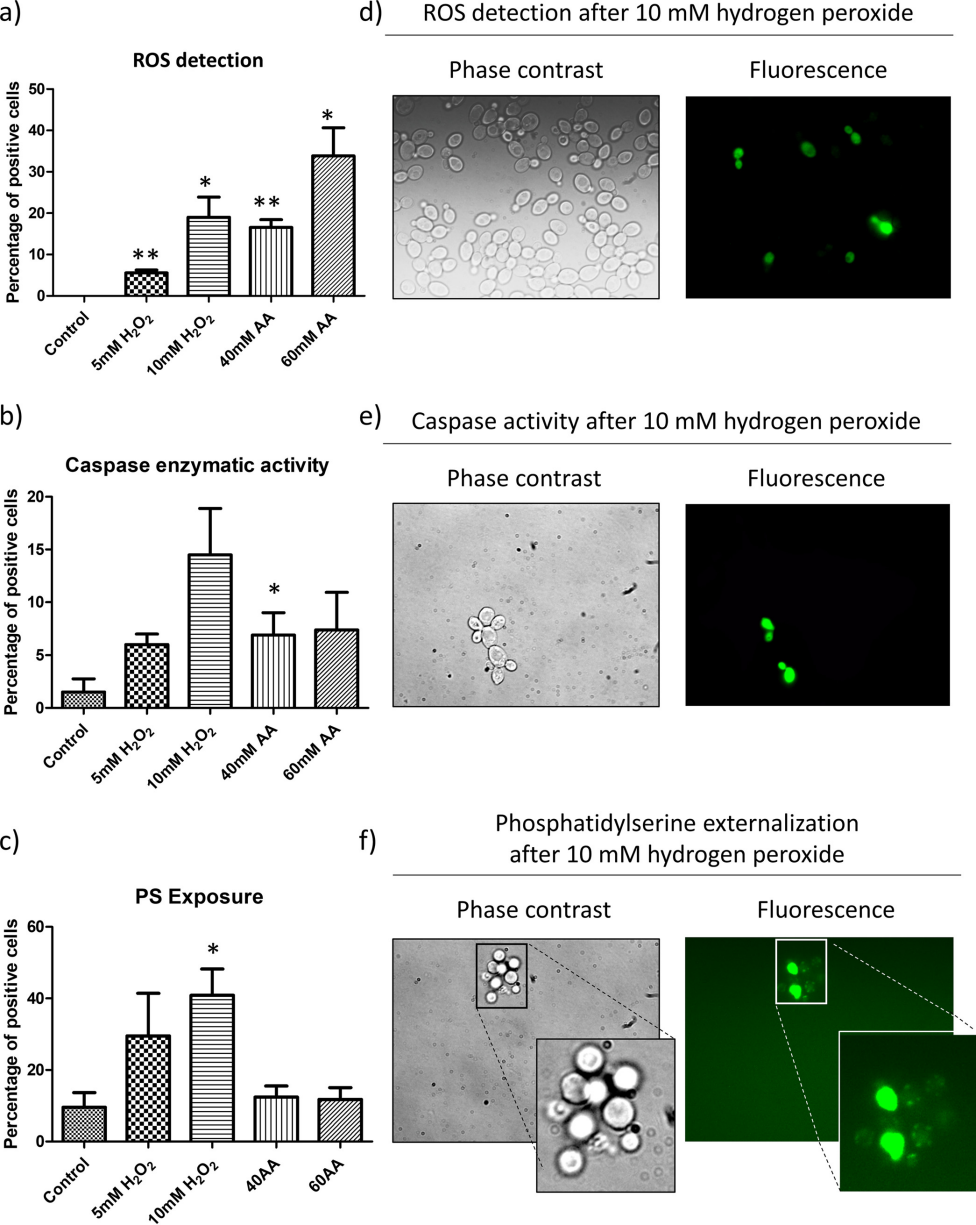
Apoptotic markers analyzed in C. albicans. (a to c) Graphics show the percentage of positive cells for ROS (a), caspase-like enzymatic activity (b), and PS exposure (c). Results represent the means of at least three biological replicates. Cells were counted on a fluorescence microscope, and significant changes are indicated (paired *t* test). (d to f) Representative fluorescence microscopy images from cells treated with hydrogen peroxide showing ROS (d), caspase activity (e), and PS exposure (f).

In order to evaluate the physiological response of C. albicans to the agents, we measured ROS accumulation and caspase-like enzymatic activity (17). Both increased after all the treatments studied, most remarkably at the highest concentrations used (Fig. 2a). ROS were detected in 20% and 34% of cells exposed to 10 mM hydrogen peroxide and 60 mM acetic acid, respectively, confirming the oxidative stress promoted by the agents. Moreover, a moderate, nonsignificant increase in caspase-like enzymatic activity demonstrated an active response of the cells to the compounds (Fig. 2b). Apoptosis evaluated by PS externalization was promoted by both concentrations of hydrogen peroxide, reaching significance after exposure to 10 mM H_2_O_2_, which resulted in up to 40% of cells becoming apoptotic (Fig. 2c). Nevertheless, the absence of a significant increase in this marker in cells exposed to acetic acid ruled out an apoptotic response under the conditions used.

### Quantitative profiling of the C. albicans proteome after exposure to stressors.

**FIG 3:**
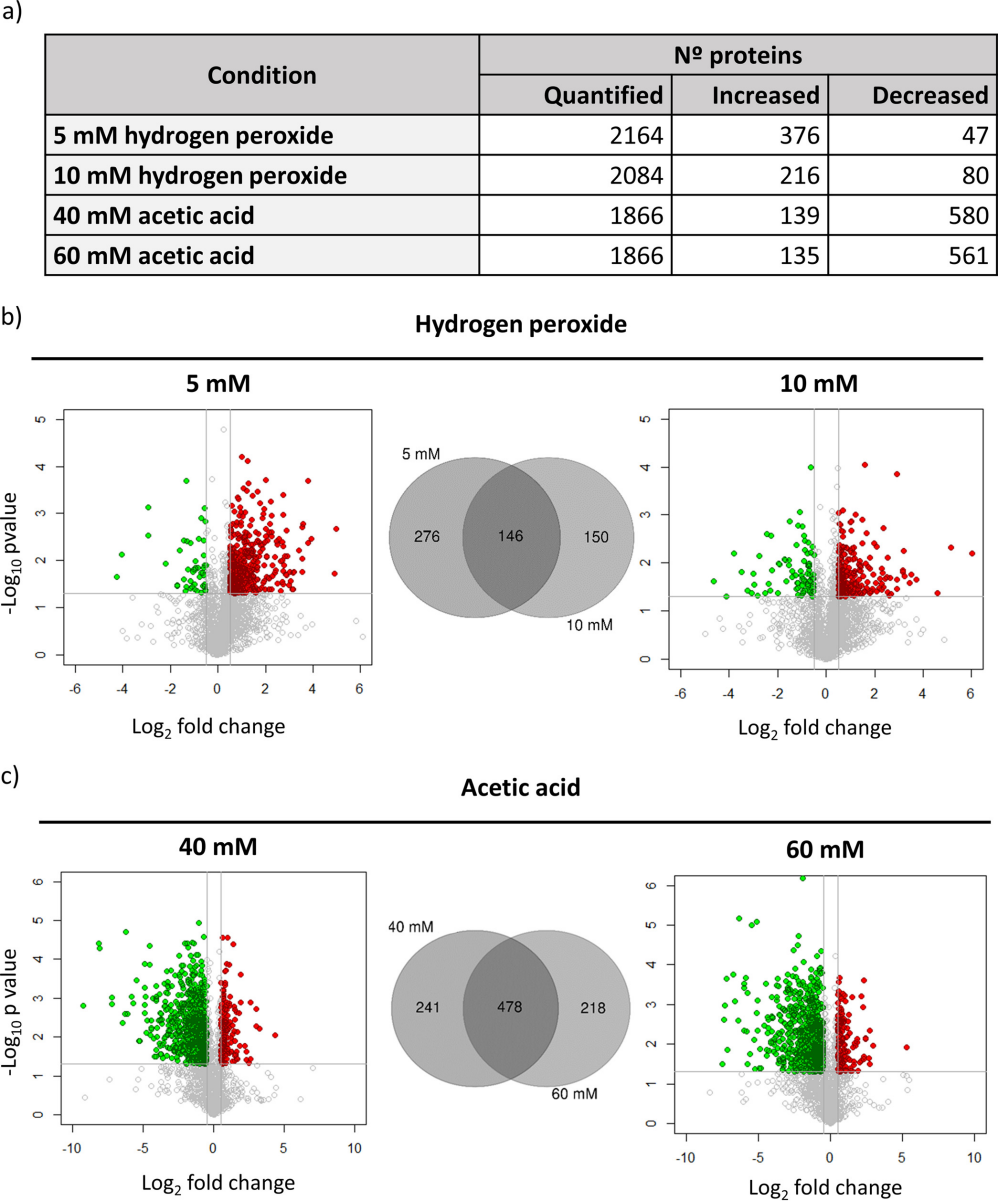
Results from DIA quantitation after exposure to hydrogen peroxide and acetic acid. (a) Number of quantified proteins and proteins with changes in their abundance under each condition. (b and c) Volcano plots represent results from DIA analysis comparing cells with and without treatment with hydrogen peroxide (b) or acetic acid (c). Changes in the abundance of proteins are represented in red or green according to their significant increase (−log_10_ *P* value, greater than 1.3; log_2_ fold change, greater than 0.5) or decrease (−log_10_ *P* value,  greater than 1.3; log_2_ fold change, less than −0.5). Venn diagrams show the number of proteins with common or specific changes at the two concentrations of hydrogen peroxide (b) and acetic acid (c) tested.

To acquire a representative picture of changes in the C. albicans global proteome after exposure to hydrogen peroxide and acetic acid, we used DIA mass spectrometry (DIA-MS). This approach allowed us to quantify more than 2,000 C. albicans proteins under the four conditions tested by using the ion library previously constructed using DDA-MS. It comprises information on almost 46.5% of the C. albicans proteome (unpublished data). Statistical analysis revealed a remarkable remodeling of the proteome, involving changes in the abundance of hundreds of proteins under each condition (Fig. 3). The proteomic responses observed after hydrogen peroxide or acetic acid treatment differed greatly. While the treatment with hydrogen peroxide predominantly promoted an increase in the abundance of a large number of proteins, the exposure to acetic acid was characterized by a profound decrease in the abundance of many proteins being more accentuated at the highest concentration of acetic acid (60 mM) (Fig. 3). These dissimilar patterns, which are clearly visible in the volcano plots in Fig. 3, are evidence of specific responses of this fungus to the two stress inducers studied.

**FIG 4:**
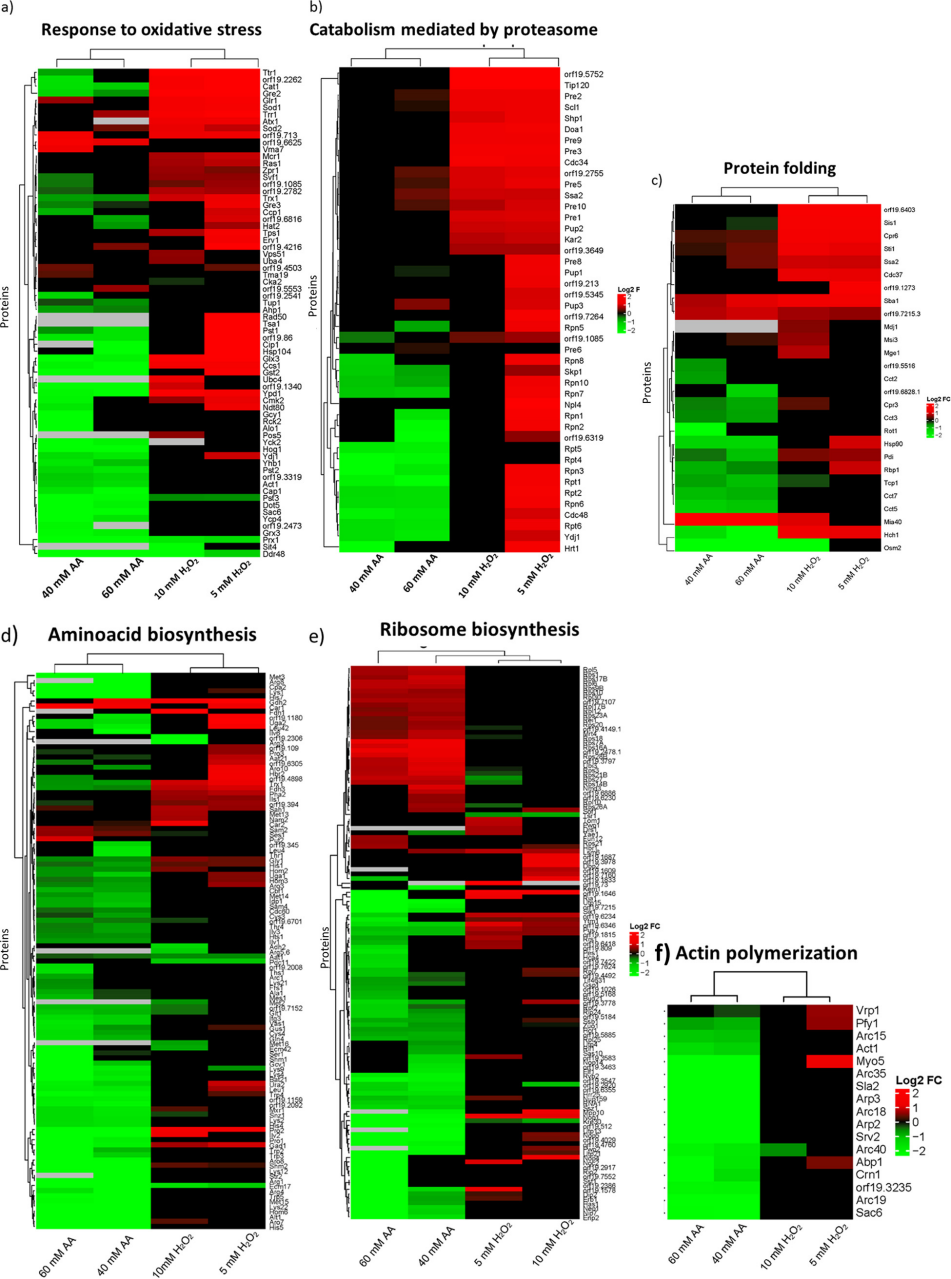
Hierarchical clustering heat maps containing proteins that changed their abundance after treatments belonging to relevant biological processes. (a) Response to oxidative stress; (b) catabolism mediated by proteasome; (c) protein folding; (d) amino acid biosynthesis; (e) ribosome biosynthesis; (f) actin polymerization. In red or green are represented proteins that increased or decreased in abundance after treatment with hydrogen peroxide (right columns) or acetic acid (left columns).

To further understand the effects of the agents on biological processes, we use Gene Ontology (GO) enrichment analysis to characterize proteins whose abundance changed significantly. Significant processes (*P* < 0.05) containing the highest number of proteins from GO enrichment analysis were represented in heat maps jointly for all treatments. This revealed an increase in key proteins related to the oxidative stress response, proteasome-dependent catabolism, and protein folding after treatment with hydrogen peroxide (Fig. 4; see also Table S1 in the supplemental material). A total of 38 and 25 proteins involved in the oxidative stress response were more abundant after treatment with 5 mM and 10 mM hydrogen peroxide, respectively. Among them were essential members of the main detoxification system in C. albicans, such as Cat1, the superoxide dismutases Sod1 and Sod2, glutaredoxin Ttr1, and the thioredoxins Tsa1, Trx1, and Trr1 (4, 23). Another relevant process unmasked by the GO analysis was cellular catabolism, with notable increases in the levels of 101 and 34 proteins after treatments with 5 mM and 10 mM hydrogen peroxide, respectively. Totals of 40 and 20 proteins involved in catabolism mediated by the proteasome were increased in abundance after treatment at the concentrations mentioned, with more proteins increasing in abundance in response to 5 mM than to 10 mM hydrogen peroxide.

10.1128/mSystems.00946-21.3 TABLE S1 GO enrichment analysis from proteins more or less abundant after treatment with 5 mM or 10 mM hydrogen peroxide. Download Table S1, XLS file, 0.2 MB. Copyright © 2021 Amador-García et al. 2021 Amador-García et al. https://creativecommons.org/licenses/by/4.0/ This content is distributed under the terms of the Creative Commons Attribution 4.0 International license.

Although the heat maps in Fig. 4 show mostly increases in most significant processes after treatments with hydrogen peroxide, other interesting groups of proteins from the respiratory chain (Cox4, Cox5, Cox6, Cox8, Cox9, and Qcr7) and cell wall (Ecm33, Pga4, Pga52, Sun41, and Tos1) had decreased levels under these conditions (Table S2).

10.1128/mSystems.00946-21.4 TABLE S2 Proteins from cell wall and involved in ATP synthesis that decrease in abundance after treatment with hydrogen peroxide. Download Table S2, XLS file, 0.05 MB. Copyright © 2021 Amador-García et al. 2021 Amador-García et al. https://creativecommons.org/licenses/by/4.0/ This content is distributed under the terms of the Creative Commons Attribution 4.0 International license.

The three biological processes with increased protein levels after treatment with hydrogen peroxide showed a completely opposite response when cells were treated with acetic acid, in which a general decrease was observed (Fig. 4). These included proteins involved in oxidative response to stress, but many heat shock proteins were also less abundant after acetic acid treatment, so the total number of proteins included in the stress response was very large. In addition, other biological processes were also characterized by a general decrease in protein levels. These up to 100 proteins participating in the biosynthesis of most amino acids were diminished in abundance after acetic acid exposure, indicating almost complete repression of this process (Fig. 4 and Table S3). Also, the actin cytoskeleton organization GO process was enriched, including proteins from the Arp2/3 family and proteins involved in actin folding (the CCT chaperone complex), which decreased in abundance after acetic acid exposure (Fig. 4). The levels of more than 30 proteins from the small and large subunits of the ribosome (in the Rps and Rpl families) increased. Conversely, the levels of 48 proteins involved in rRNA processing (e.g., Nop5, Rrs1, Utp13, Utp15, and Utp21) declined.

10.1128/mSystems.00946-21.5 TABLE S3 GO enrichment analysis from proteins more or less abundant after treatment with 40 mM or 60 mM acetic acid. Download Table S3, XLS file, 0.6 MB. Copyright © 2021 Amador-García et al. 2021 Amador-García et al. https://creativecommons.org/licenses/by/4.0/ This content is distributed under the terms of the Creative Commons Attribution 4.0 International license.

### Opposite impacts on proteasome activity after hydrogen peroxide and acetic acid treatment in C. albicans.

Our proteomic approach demonstrated that hydrogen peroxide and acetic acid treatments had opposite effects on the abundance of proteasome proteins. The proteasome is responsible for the specific degradation of abnormal, short-lived, and regulatory proteins and comprises a central catalytic component (20S) with three major proteolytic activities—chymotrypsin-like, trypsin-like, and peptidyl-glutamyl peptide hydrolyzing activities—and a regulatory particle (19S) conferring ATP and ubiquitin dependence on protein degradation (24).

**FIG 5:**
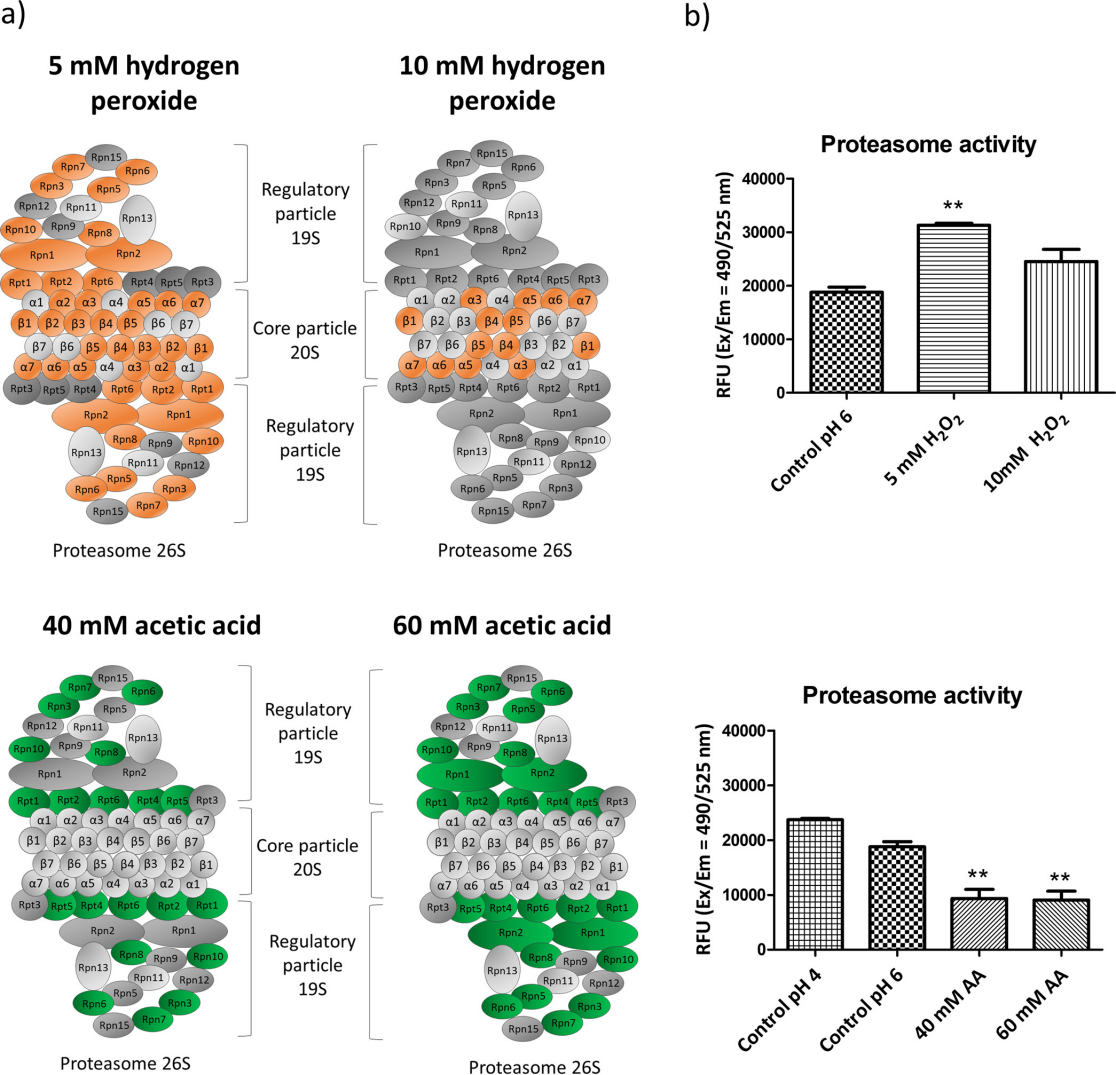
Changes in the abundance of proteins from the proteasome after exposure to acetic acid or hydrogen peroxide. (a) In orange or green are highlighted proteins that increased or decreased significantly. (b) Results from the measurement of chymotrypsin-protease activity of the proteasome in relative fluorescence units (RFU).

Our data showed an increase in the expression levels of proteasome subunits after hydrogen peroxide treatment. This was more noticeable after 5 mM hydrogen peroxide treatment, with increased levels of 21 out of 25 quantified proteasome proteins representing both the regulatory and the central core particle (Fig. 5a). After 10 mM hydrogen peroxide treatment, only seven proteins from the catalytic particle were more abundant (Pre1, Pre2, Pre3, Pre5, Pre9, Pre10, and Pup2). In contrast, a dramatic decrease in proteasome protein levels occurred after 40 mM and 60 mM acetic acid treatment, being greater with the higher concentration. Surprisingly, this affected only proteins from the regulatory particle (Rpn1, Rpn2, Rpn3, Rpn5, Rpn6, Rpn7, Rpn8, Rpt1, Rpt2, Rpt4, Rpt5, and Rpt6) (Fig. 5a).

In order to correlate these changes in the abundance of proteasome proteins to differences in proteasome activity, the chymotrypsin-like protease activity associated with the proteasome complex was measured. The results entirely corroborate our proteomic findings. The exposure to hydrogen peroxide increased proteasome activity, while the acetic acid treatment caused a loss of activity. To ensure that this effect was not related to the drop in pH caused by acetic acid treatment, a control sample acidified by HCl was also tested and the decrease in proteasome activity was not observed. In addition, the dose-response effect observed by proteomics in relation to the two concentrations of hydrogen peroxide was also correlated with proteasome activity, which increased by 67% after 5 mM hydrogen peroxide treatment but by only 31% after 10 mM hydrogen peroxide treatment with respect to the control (Fig. 5b). The treatment with acetic acid also revealed the dose-dependent effect leading to decreases in the proteasome activity of 48% and 50% after treatment with 40 mM and 60 mM, respectively. This decrease in proteasome activity presumably led to an accumulation of ubiquitinated proteins which was also confirmed in this work by their detection by Western blotting (Fig. S1).

10.1128/mSystems.00946-21.2 FIG S1 Protein ubiquitination in response to the treatment with different doses (40 and 60 mM) of acetic acid (AA). Download FIG S1, PDF file, 1.2 MB. Copyright © 2021 Amador-García et al. 2021 Amador-García et al. https://creativecommons.org/licenses/by/4.0/ This content is distributed under the terms of the Creative Commons Attribution 4.0 International license.

### Proteomic analysis unmasks proteins highly relevant to oxidative stress.

Focusing on the top 10 proteins with the greatest increase in abundance after treatment with both concentrations of hydrogen peroxide (Table 1), we found proteins associated with the proteasome (Hsm3, orf19.5752, and Tip120) and response to oxidative stress (Gre2, Oye2, and Oye23). Also among those with the most prominent increases in expression were proteins involved in DNA repair (Hsm3), DNA replication (Mcm2), and iron and copper metabolism (orf19.2067 and Cup1).

**TABLE 1:** List of the 10 proteins showing the highest increase in relative abundance after treatment with hydrogen peroxide

Identifier	Protein	Description	Biological process(es)	Hydrogen peroxide			
				5 mM		10 mM	
				Ratio log_2_	*P* value	Ratio log_2_	*P* value
orf19.3150	Gre2	Putative reductase	Response to oxidative and osmotic stress	4.97	0.00	4.60	0.04
orf19.2467	Prn1	Protein with similarity to pirins	Unknown	4.90	0.02	5.15	0.00
orf19.6729	Tip120	Protein involved in regulation of SCF^*a*^	Proteasomal catabolic process	3.58	0.00	3.16	0.02
orf19.5752		Protein involved in regulation of SCF	Proteasomal catabolic process	3.56	0.02	3.45	0.03
orf19.3443	Oye2	NAPDH dehydrogenase	Oxidation reduction process, apoptosis	3.55	0.00	3.15	0.01
orf19.3433	Oye23	NADPH dehydrogenase	Oxidation reduction process	3.54	0.01	3.23	0.02
orf19.2067		Mitochondrial iron metabolism	Iron ion binding	3.40	0.01	3.19	0.01
orf19.3940.1	Cup1	Metallothionein; copper resistance	Copper ion binding	3.20	0.02	2.93	0.03
orf19.1331	Hsm3	Proteasome regulatory particle	Proteasome regulation and mismatch repair	2.92	0.01	2.59	0.05
orf19.4354	Mcm2	Phosphorylated protein of unknown function	DNA replication	2.87	0.02	2.93	0.02

a SCF, complex containing Skp, Cullin, and F-box.

**FIG 6:**
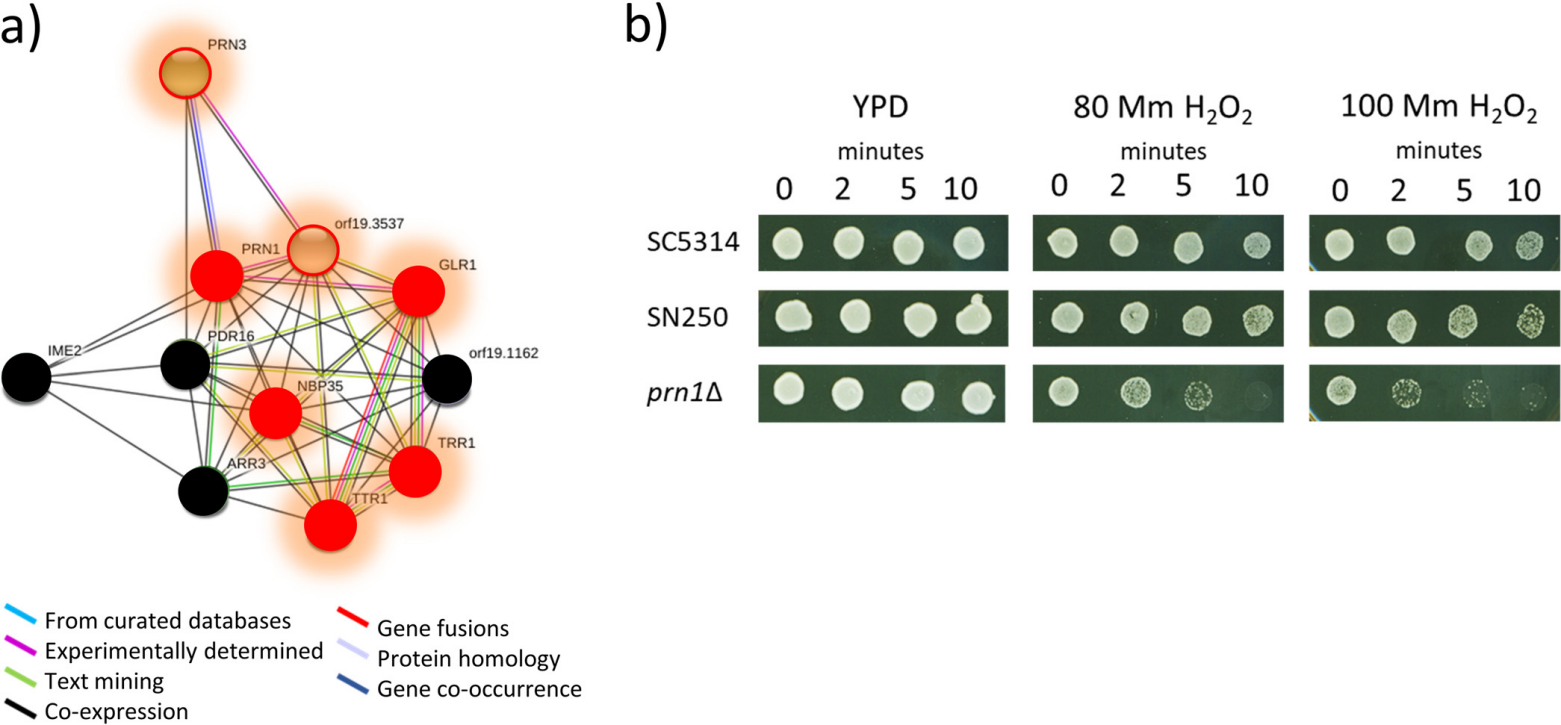
Prn1 protein in oxidative stress. (a) Network showing predicted functional partners of Prn1 according to String software. In red are proteins that increased in abundance after exposure to hydrogen peroxide in this work. Proteins circled in red were quantified only after hydrogen peroxide exposure. In black are proteins that were not detected. (b) Sensitivity to 80 and 100 mM hydrogen peroxide of the wild-type strains SC5314 and SN250 compared to the *prn1*Δ mutant.

The increase in the level of Prn1, a protein similar to pirins, whose function, biological process, and cellular component in C. albicans remain unknown, was particularly interesting. This protein exhibited the highest increase after treatment with 10 mM hydrogen peroxide and the second highest after treatment with 5 mM hydrogen peroxide. Furthermore, Prn1 was the only protein with a greater increase after treatment with 10 mM than with 5 mM hydrogen peroxide. This result suggests a main role for Prn1 in the response to oxidative stress. Analysis of predicted functional partners of this protein by String software showed networks with other key proteins in oxidative stress. In addition, most of them also increased in abundance in our experiments (Fig. 6a). To phenotypically validate this, we analyzed the susceptibility of a *prn1*Δ mutant to hydrogen peroxide in comparison with wild-type strains. As shown in Fig. 6, the susceptibility of the *prn1*Δ mutant to 80 mM and 100 mM hydrogen peroxide was notably higher than those of the strain used in this work (SC5314) and the control strain from the Noble collection (SN250) (Fig. 6b).

### The SRM method for quantitation of apoptotic proteins after exposure to stress inducers.

Global proteomic results provide a panoramic picture of protein changes, but we were also very interested in specific proteins involved in regulated cell death. We chose targeted proteomics as the most useful and efficient means of analyzing them. We developed an SRM method to study these proteins. First, we selected 32 C. albicans proteins previously described (for C. albicans or S. cerevisiae) as relevant to yeast apoptosis involving mitochondria, the Ras pathway, or Mca1 (Table 2) (8, 14–16, 18, 25–54). SRM quantitation of 63 peptides and 462 transitions was performed on samples from C. albicans exposed to 5 mM or 10 mM hydrogen peroxide and 40 mM or 60 mM acetic acid (Table S4). Of the 32 proteins included in the SRM method, 22 were quantified under at least one condition. Comparison of DIA and SRM quantitation showed that some proteins were quantified by only one proteomic approach. The levels of proteins that were quantified in both the global DIA and the targeted SRM analyses were compared. In most cases, the two methods showed similar changes in protein levels (Table 3). In some cases the changes were not significant for one of the techniques, and in only two cases was the trend opposite, indicating that we should be especially cautious with the peptides selected and use more peptides to ensure a more accurate quantification by the targeted approach of these proteins. The targeted approach revealed changes in the abundance of dozens of the selected proteins under most of the conditions tested, confirming the importance of these proteins upon exposure to these agents (Table 4). Results from the SRM quantitation showed a global pattern similar to that observed in the DIA analysis, with increased and decreased levels of many proteins selected after hydrogen peroxide and acetic acid exposure, respectively.

**TABLE 2:** Proteins involved in C. albicans or S. cerevisiae apoptosis selected for SRM analysis^*a*^

C. albicans	S. cerevisiae	Description	Apoptosis^*b*^	Reference(s)
Mitochondria				
Cpd1	Aif1	Mitochondrial apoptosis-inducing factor	Pro	14, 15, 35, 38, 53
Cpr3	Cpr3	Putative peptidyl-propyl *cis-trans* isomerase	Pro	33, 35
Cyc1	Cyc1	Cytochrome *c*	Pro	15, 37, 40
Dnm1	Dnm1	Putative dynamin-related GTPase	Pro	15, 28
Nde1	Nde1	Putative NADH dehydrogenase	Pro	15, 35
Nuc1	Nuc1	Major mitochondrial nuclease	Pro	15, 38
orf19.4423	Ysp2	Mitochondrial glucosyltransferase	Pro	15, 43
orf19.916	Bxi1	Protein involved in apoptosis	Anti	26, 27
Oye2	Oye2	NAPDH dehydrogenase	Anti	29, 41
Oye32	Oye3	NAD(P)H oxidoreductase	Pro	41, 52
Pet9	Pet9	Mitochondrial ADP/ATP carrier protein involved in ATP biosynthesis	Pro	42
Por1	Por1	Mitochondrial outer membrane porin	Pro/anti	29, 34, 42, 54
Rsm23	Rsm23	Mitochondrial ribosomal subunit	Pro	25
Sod2	Sod2	Mitochondrial superoxide dismutase	Anti	15
Tma19	Tma19	Translation machinery associated	Pro	15, 45
Ymx6	Nde1	NADH dehydrogenase	Pro	15, 32, 35
Ras/PKA/AMPc				
Bcy1	Bcy1	Protein kinase A regulatory subunit	Anti	8
Cyr1	Cyr1	Class III adenylyl cyclase		16
Efg1	Sok2	bHLH^*c*^ transcription factor	Anti	49
Ras1	Ras2	RAS signal transduction GTPase	Pro	8, 14
Tpk1	Tpk1	cAMP-dependent protein kinase catalytic subunit	Pro	8
Mca1 and substrates				
Cdc48	Cdc48	Putative microsomal ATPase	Anti	14, 18, 31
Mca1	Mca1	Putative metacaspase involved in apoptosis	Pro	14, 31, 39
Nma111	Nma111	Putative serine protease	Pro	15, 29, 38, 50, 51
orf19.643	Bir1	Orthologues have role in apoptosis	Anti	15, 29, 38, 51
Tdh3	Tdh3	Glyceraldehyde-3-phosphate dehydrogenase	Pro	46
Wwm1	Wwm1	Protein of unknown function	Pro	31, 47
Others				
Cst20	Ste20	Protein kinase	Pro	15
orf19.2541	Tat-D	Protein with endonuclease activity	Pro	15, 44
orf19.5943.1	Stm1	Protein of unknown function	Anti	34, 36
Rny11	Rny1	Protein with endonuclease activity	Pro	26, 48
Svf1	Svf1	Putative survival factor	Anti	30, 33

a Proteins are related to regulated cell death by mitochondria, the Ras/PKA/AMPc pathway, metacaspase 1 (Mca1), or other pathways.

b Pro, proapoptosis; anti, antiapoptosis.

c bHLH, basic helix-loop-helix.

**TABLE 3:** Results from SRM and DIA comparison from the protein set that confirmed the SRM method

Protein	Result^*a*^ with:			
	Hydrogen peroxide		Acetic acid	
	5 mM	10 mM	40 mM	60 mM
Bcy1	✓	✓	✓	✓
Cdc48	✓	X	✓	✓
Cpr3	DIA	DIA	✓	✓
Cyc1	X	X	✓	X
Efg1	∼	∼	XX	✓
Mca1	✓	X	✓	X
Nde1	-	-	-	SRM
Nma111	DIA	DIA	X	X
Nuc1	X	X	X	XX
orf19.4423	-	-	SRM	SRM
orf19.5943.1	X	X	X	∼
orf19.643	-	-	SRM	SRM
orf19.916	-	-	SRM	SRM
Oye2	✓	✓	X	X
Oye32	✓	∼	X	X
Pet9	DIA	DIA	DIA	DIA
Por1	✓	X	X	✓
Ras1	✓	X	∼	X
Rsm23	DIA	DIA	✓	DIA
Svf1	✓	✓	✓	∼
Tdh3	∼	∼	✓	X
Tma19	X	X	✓	✓

a ✓, same result in DIA and SRM; “∼” same fold change trend in DIA and SRM but with *P* value close to significance in one of the strategies; X, nonsignificant change in one of the strategies; XX, opposite fold change by DIA and SRM; -, unable to quantify by DIA or SRM; SRM, quantified only by SRM; DIA, quantified only by DIA. The comparison was performed taking into account significant *P* values of <0.05 but without a threshold in ratio (ratio > 0 or ratio < 0).

**TABLE 4:** Results from SRM quantitation of proteins involved in regulated cell death

Pathway and protein	Result^*a*^ with:							
	Hydrogen peroxide (mM)		Acetic acid (mM)			Amphotericin B (μg/ml)		
	5	10	40	60	120	1	2	4
Mca1								
Cdc48	0.5	0.8	−1.8	−1.7	ns	−0.4	NS	NS
Mca1	NS	−0.5	−1.7	−1.0	−0.5	-0.9	NS	NS
Nma111	—	—	−0.9	−0.7	NS	NS	NS	NS
orf19.643	—	—	−1.0	−1.2	—	—	—	—
Tdh3	−0.5	−0.8	−1.1	NS	NS	NS	NS	NS
Mitochondria								
Cpr3	NS	NS	−1.9	−2.5	0.3	NS	NS	NS
Cyc1	−1.8	−1.3	2.0	1.5	NS	0.7	NS	NS
Nde1	—	—	—	—	NS	0.6	NS	NS
Nuc1	−2.6	−0.7	−0.4	−0.7	NS	NS	NS	NS
orf19.4423	—	—	−0.6	NS	—	—	—	—
orf19.916	—	—	NS	NS	—	—	—	—
Oye2	2.0	2.1	−0.9	−1.4	NS	NS	NS	NS
Oye32	0.7	1.4	NS	−0.3	2.6	2.3	NS	NS
Pet9	—	—	—	—	NS	NS	NS	—
Por1	ns	0.4	0.3	NS	NS	NS	NS	NS
Rsm23	—	—	NS	—	NS	NS	NS	NS
Tma19	−1.0	−0.6	0.2	NS	NS	NS	NS	NS
Other								
orf19.5943.1	−0.5	−0.2	0.3	0.4	NS	NS	NS	NS
Svf1	0.6	0.5	−0.3	−1.0	NS	0.4	NS	NS
Ras1								
Bcy1	NS	NS	−0.4	−1.0	NS	0.1	NS	NS
Efg1	−1.1	NS	0.9	-1.0	−0.7	−0.3	NS	NS
Ras1	0.5	NS	−0.5	−0.4	NS	NS	NS	NS

a Positive and negative log_2_ values correspond to a significant (*P* value < 0.05) increases and decreases in the abundance of proteins, respectively. NS, no significant change (*P* value > 0.05). —, not quantified by SRM under the condition indicated.

10.1128/mSystems.00946-21.6 TABLE S4 SRM method. Download Table S4, XLS file, 0.1 MB. Copyright © 2021 Amador-García et al. 2021 Amador-García et al. https://creativecommons.org/licenses/by/4.0/ This content is distributed under the terms of the Creative Commons Attribution 4.0 International license.

**FIG 7:**
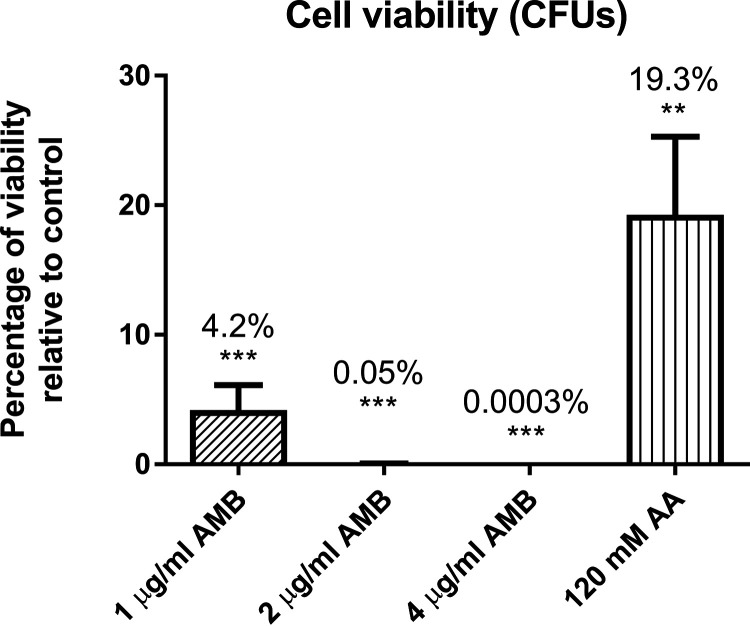
Percentage of viable cells after treatments with several concentrations of amphotericin B or 120 mM acetic acid compared to control samples. A significant change is indicated as follows: **, *P* value < 0.01, and ***, *P* value < 0.001 (paired *t* test). Results represent the means of at least three biological replicates.

We use the SRM method created to measure the participation of these proteins in C. albicans exposed to 1, 2, and 4 μg/ml of AMB and 120 mM acetic acid, conditions previously described as apoptotic (16, 22). We previously confirmed that all these conditions led to a loss of viability but not to a high percentage of loss of membrane permeability (always less than 1%, except less than 8% for 4 μg/ml AMB) (Fig. 7). The SRM analysis revealed changes in the abundance of proteins at 1 μg/ml AMB and 120 mM acetic acid. There were no significant changes at higher doses of AMB (2 and 4 μg/ml) (Table 4).

**FIG 8:**
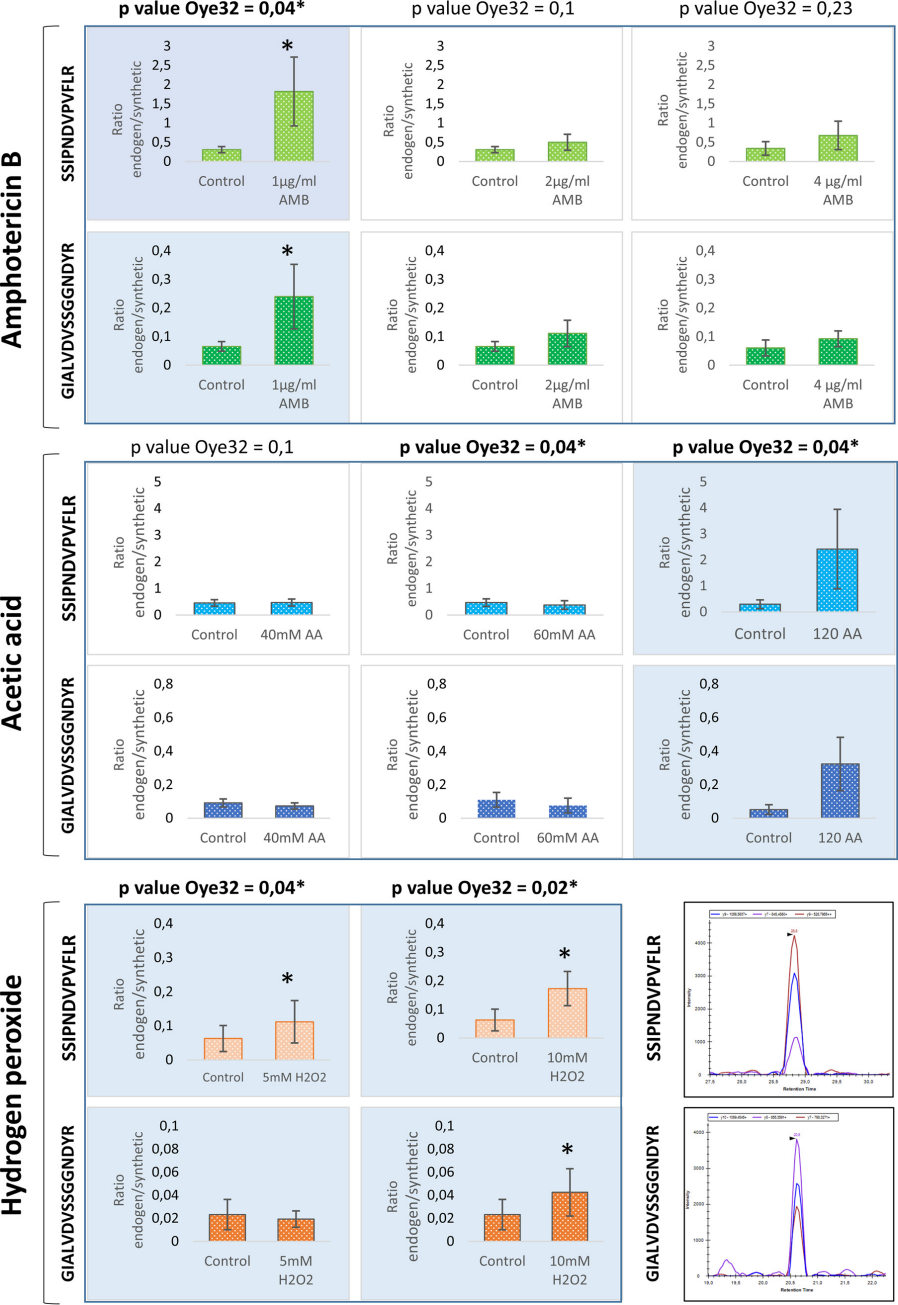
Quantification of peptides from Oye32 protein by SRM after treatment with AMB, acetic acid, or hydrogen peroxide. Blue shade in graphs indicate the conditions where apoptosis was demonstrated by PS exposure and an increase in Oye32 protein was observed (*). Note that the intensity of the peptide GIALVDVSSGGNDYR at 5 mM H_2_O_2_ in some replicates was too low to find significant differences from the control. In the lower right corner is shown a representative chromatogram from the two peptides of Oye32 quantified by SRM.

**FIG 9:**
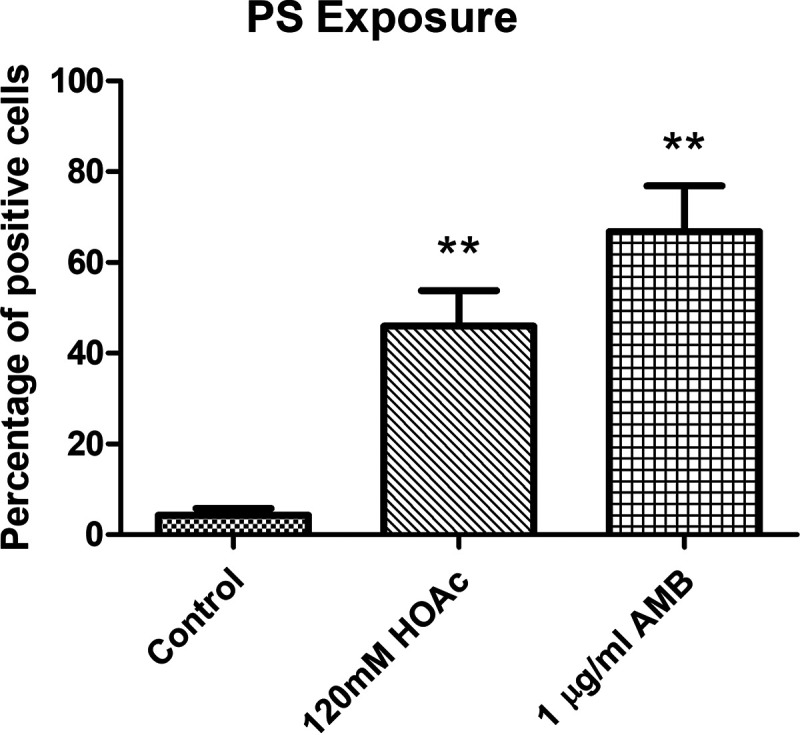
Percentage of cells showing PS exposure after treatments with 1 μg/ml amphotericin B or 120 mM acetic acid compared to control samples. A significant change is indicated as follows: **, *P* value < 0.01 (paired *t* test). Results represent the means of at least three biological replicates.

Interestingly, the protein Oye32 increased in abundance upon treatment with 120 mM acetic acid and 1 μg/ml AMB (Fig. 8), as it did in response to treatment with 5 mM and 10 mM hydrogen peroxide. In view of these results, we wished to determine whether these increases in Oye32 abundance were also correlated with an apoptotic state. Apoptosis occurred in 46% and 67% of cells treated with 120 mM acetic acid and with 1 μg/ml AMB, respectively (Fig. 9). These results place Oye32 in the spotlight as a common marker of C. albicans apoptosis induced by multiple stressors.

## DISCUSSION

**FIG 10:**
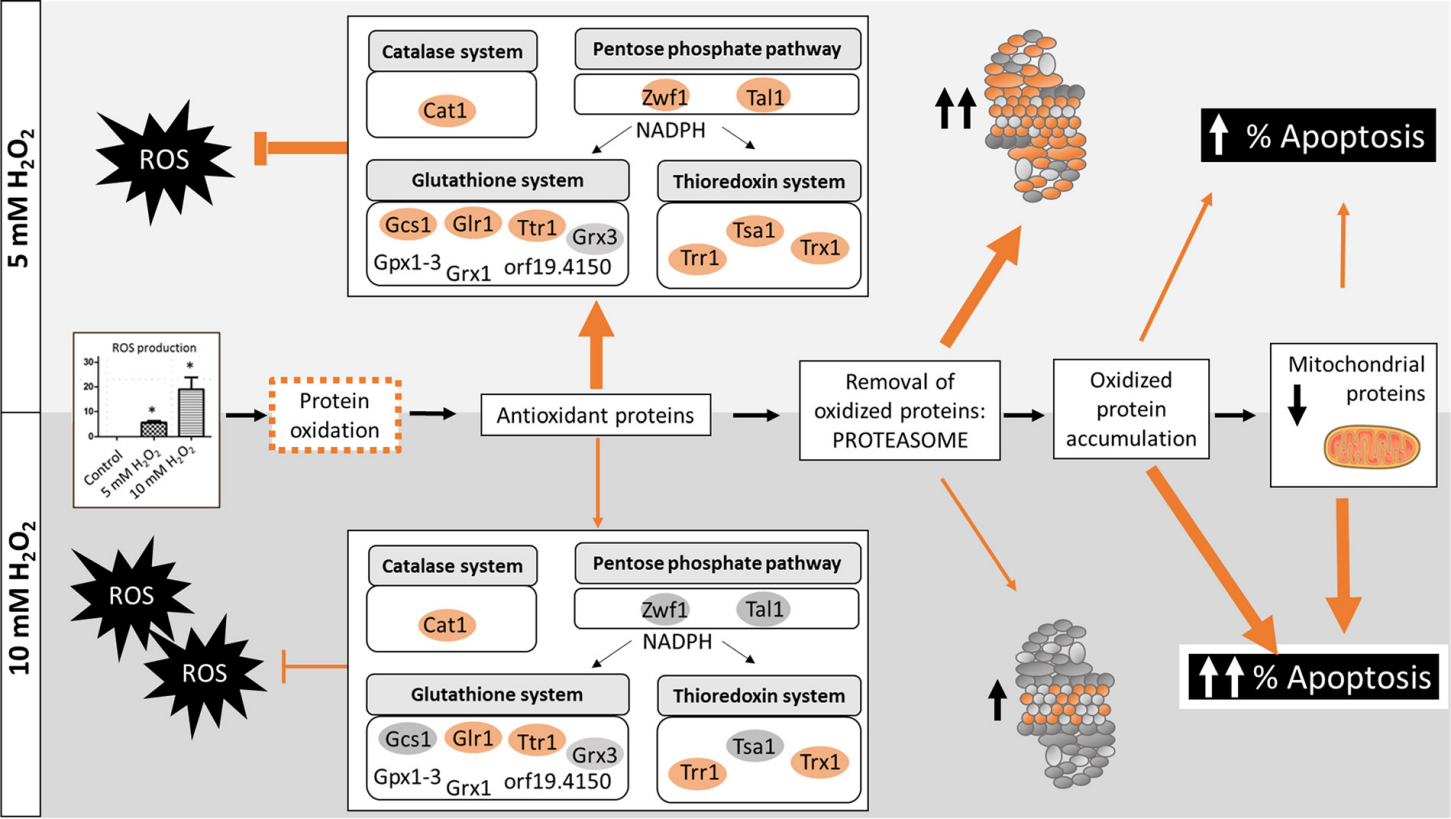
Global overview of C. albicans response to hydrogen peroxide according to the results from this work. Oxidative damage in proteins started with the increase in ROS accumulation, which triggered an antioxidant response with the increase of proteins from main detoxification systems (catalase, glutathione, and thioredoxin). The increase of antioxidant proteins was less remarkable than at the higher concentration of hydrogen peroxide (10 mM) leading to the accumulation of oxidized proteins. This effect joined to the lower activity of the proteasome observed at 10 mM than at 5 mM hydrogen peroxide led to mitochondrial damage and an increase in apoptosis percentage in 10 mM hydrogen peroxide. The thicknesses of lines indicate the activation of each process. Adapted from the work of Costa et al. (57).

We have enlarged the map of the C. albicans global proteomic response to hydrogen peroxide and acetic acid as representative stressors in the host. The high-throughput DIA-MS proteomic analysis is one of the most extensive characterizations of C. albicans proteome remodeling in response to these conditions to date (52, 55). The analysis also provides a global vision of the proteome upon apoptosis induced by hydrogen peroxide in C. albicans. Both concentrations of hydrogen peroxide assayed (5 mM and 10 mM) stimulated apoptotic cell death in C. albicans, as demonstrated by PS exposure and accompanied by an increase in ROS accumulation and caspase-like enzymatic activity. In this context, the proteomic results revealed increases in the abundance of proteins involved in antioxidant defense systems, proteasome-mediated catabolism, and protein folding, indicating an active response of the fungus against the agent. The increase in antioxidant proteins such as Cat1, Sod2, Tsa1, Trr1, Glr1, and Glx3 is consistent with previous proteomic and transcriptomic studies performed on C. albicans after hydrogen peroxide exposure (52, 55, 56). However, our study by DIA-MS provide a more detailed picture of the cell (46.5% of proteins quantified), unraveling changes in several hundred proteins, in contrast to previous works that revealed changes in dozens of proteins (55, 56). This allows, for instance, description of up to 40 proteins involved in proteasome degradation, while previous works using other approaches describe only 3 proteins at the same concentration of hydrogen peroxide (55). The proteasome plays an essential role in the removal of oxidatively damaged proteins, so the results are consistent with the environmental insult promoted by hydrogen peroxide. Moreover, proteins involved in protein folding were also increased in abundance, consistent with a need to repair oxidatively damaged proteins. Intriguingly, a higher dose of hydrogen peroxide was more effective in inducing apoptosis and resulted in increased levels of fewer proteins belonging to the highlighted biological processes. This result suggested a failure in the proteomic response of cells upon more severe treatment. The minor antioxidant response observed after exposure to 10 mM hydrogen peroxide, including key elements of this response, could be what induced apoptosis in a larger percentage of cells (Fig. 10). Our proteomic results reflect the same scheme of oxidative damaged as proposed by Costa et al. for S. cerevisiae (57). According to Costa and colleagues, an increase in ROS will lead to the oxidation of proteins, which can be repaired by antioxidant systems or degraded by the proteasome if irreversibly damaged. Extensively oxidized proteins can form aggregates that cannot be degraded, impairing 20S proteasome and mitochondrial function. Our results demonstrate a reduction in proteasome activity in 10 mM compared with 5 mM hydrogen peroxide. Furthermore, some mitochondrial proteins from the respiratory chain, such as Cox4, Cox5, Cox6, Cox8, Cox9, and Qcr7, were also less abundant after the treatment (57).

In addition, our proteomic study uncovered the relevance of Prn1, a protein with unknown function in C. albicans that, according to our results, protects the cell from oxidative stress. This is supported by the increase in sensitivity to oxidative stress of a Prn1 deletion mutant. Prn1 is similar to the pirins, proteins that have been related to cellular stress and whose overexpression has been linked to apoptosis in multiple kingdoms (58, 59). For instance, in Streptomyces ambofaciens, a protein similar to the pirins has been associated with resistance to oxidative stress (60). Our results are the first evidence of a role for Prn1 in the response to oxidative stress in C. albicans. This protein lacks a homologue in S. cerevisiae, suggesting that it may be specific to pathogenic fungi. This crucial finding highlights the usefulness of global proteomic analysis in revealing the functions of unknown proteins.

Global proteomic analysis of C. albicans cells subjected to acetic acid stress provided detailed information about the main cellular components that are altered in response to a fungistatic agent. Few large-scale studies have focused on the response of C. albicans to acetic acid; some of them have analyzed the transcriptome exclusively, and most of them have been performed on S. cerevisiae for its interest to industry (61–64). Our results showed a dramatic decrease in the abundance of numerous proteins involved in amino acid biosynthesis, oxidative stress, protein folding, the proteasome, actin polymerization, and translation, correlated with arrested cell growth. Our results agree with previous results for S. cerevisiae demonstrating a block in amino acid biosynthesis, which could be favorable for economy in energy (64, 65). Despite the ROS accumulation in cells upon acetic acid insult, the abundance of many proteins related to oxidative stress protection declined, a phenomenon previously described for S. cerevisiae (63, 66). Some authors have suggested that an increase in ROS upon acetic acid exposure could somehow aid the cell’s response (65). Our results would support that the ROS detected could be a consequence of a mitochondrial impairment, as we observed some decreased proteins from the electron transport chain Mci4, Sdh1, Sdh2, Cox6, Atp1, Atp3, Vma2, Vma4, Vma5, Vma8, and Vma13.

Surprisingly, ribosomal subunit proteins were notably more abundant upon acetic acid treatment, in contrast with the general decrease observed in proteomic pattern. In accordance with these results, Cottier et al. showed previously an upregulation of genes related to ribosomal biogenesis (62). Furthermore, the decreased abundance of the proteasome subunits and consequent loss of proteasome activity could lead to an accumulation of ribosomal proteins usually produced in excess by cells (67). Ribosomal proteins are among the most prominent ubiquitin-conjugated species that accumulate upon proteasome inhibition. Consequently, the decline in proteasome activity caused by acetic acid could explain the increase in the abundance of up to 30 proteins from the large and small ribosomal subunits. In addition, despite the presence of large amounts of ribosomal subunit proteins, the observed reduction in levels of proteins that are crucial for accurate rRNA processing could prevent the efficient formation of mature ribosomes and thus interfere with translation (68).

Some key cellular components responded to various agents in opposite manners. While the increase in proteasome subunits after exposure to hydrogen peroxide would be protective, the decrease in the abundance of proteasome proteins upon acetic acid treatment and consequent inhibition of proteasome activity could, presumably, cause ribosomal subunits to accumulate.

This study included a detailed proteomic characterization of the behavior of subunits from the core and regulatory particles of the proteasome. The functional validation of the proteomic data linked the detected changes in the amounts of proteins to changes in proteasome activity. The proteasome has been assigned a dual role in regulated cell death (69). Some proteasome inhibitors are attracting interest for their potential therapeutic use (70). For instance, bortezomib, a proteasome inhibitor used in cancer therapies, enhances the activity of fluconazole in antifungal treatment (71). In light of our results, the slight increase in proteasome activation would affect the cell to counterbalance the oxidative stress leading to apoptotic cell death.

Unlike global proteomics, a targeted proteomic approach can focus on relevant processes within the cell, such as apoptosis. Similar results obtained by the two methods support the validity of the observed changes. The different results from the two methods observed for some proteins could be explained by the use of different peptides in the quantification as well as the distinct sensitivities of the equipment used for global and targeted approaches. In these cases, the change in the abundance should be considered with caution and based on quantotypic peptides in the SRM method, where the number of peptides used is limited (72). The SRM method could be improved by using peptides that were efficiently detected and quantified in the DIA approach. Moreover, some proteins that could only be quantified using the SRM method demonstrated that the integration of the two approaches contributes to novel results.

Cells treated with hydrogen peroxide exhibited an increase in relevant proteins such as Oye2 and Oye32, oxidoreductases described in S. cerevisiae for their role in apoptosis (41) (Table 4). Furthermore, the observed increase in Ras1 abundance is in accord with a prior study of apoptosis induced by hydrogen peroxide (16). The increase in Svf1 (a putative survival factor), which is crucial for the survival of cells under oxidative stress, revealed the effort of cells to overcome the stress. The increase in Cdc48 was closely related to proteasome activity, and the decrease in mitochondrial proteins such as Cyc1, Nuc1, and Tma19 also reflected the alteration of these cellular components previously unmasked by the global proteomic analysis using DIA-MS. Overall, these results support the relevance of these proteins to apoptotic cell death induced by 5 mM and 10 mM hydrogen peroxide and reinforce the use of the SRM method to evaluate the roles of proteins in apoptosis.

Despite the absence of an apoptotic phenotype (PS exposure) in cells treated with acetic acid, we used the SRM method to study the behavior of the selected proteins under stress. The SRM results were consistent with the findings of our DIA-MS global proteomics analysis: the decline in the levels of most proteins in the SRM panel was evidenced for the arrested state of cells treated with acetic acid.

The targeted proteomic approach revealed a consistent increase in the abundance of Oye32 in cells subjected to various stresses that can induce apoptosis (5 mM and 10 mM hydrogen peroxide, 120 mM acetic acid, and 1 μg/ml AMB), suggesting this protein as a possible apoptotic biomarker.

The SRM method designed in this study is a straightforward proteomic approach to monitoring apoptotic proteins that might be targets for antifungal therapies in several circumstances. The existence of regulated cell death in fungal pathogens could be usefully exploited to develop novel antifungal therapies (6, 73).

## MATERIALS AND METHODS

### Fungal strains and culture conditions.

Candida albicans wild-type strain SC5314 was used for the phenotypic and proteomic analyses performed. For hydrogen peroxide susceptibility assays, control strain SN250 and the *prn1* mutant from the Noble collection were used (74). Yeast cells were maintained at 30°C on YPD medium containing 1% yeast extract, 2% peptone, and 2% glucose with rotatory shaking (180 rpm). Before exposure to agents, cells were grown until exponential phase was reached (optical density, 1 ± 0.2). Then acetic acid, hydrogen peroxide, or amphotericin B (AMB) was added to the desired concentration and incubated for 200 min at 30°C with rotatory shaking. For the phenotypic and proteomic assays, after treatment with hydrogen peroxide final concentrations of 5 mM and 10 mM were selected (Sigma-Aldrich). For susceptibility assays, C. albicans strains grown at an optical density of 1 were exposed to 80 and 100 mM hydrogen peroxide for 0, 2, 5, or 10 min. Four microliters of yeast suspension was spotted onto YPD agar plates and incubated for 48 h at 30°C.

The acetic acid concentrations used in the experiments were 40, 60, and 120 mM (PanReac; AppliChem). For AMB assays, a stock solution (1 mg/ml) was prepared in dimethyl sulfoxide (DMSO; amphotericin B, 85%; Acros Organics, Fisher). AMB was added to an exponential-phase culture at final concentrations of 1, 2, and 4 μg/ml for 200 min at 30°C in a rotatory shaker.

### Viability assays.

To determine the percentage of viable cells after suspension of the treatment, cells were collected and washed three times with phosphate-buffered saline (PBS). Optical densities (620 nm) were measured to normalize the amount of cells and correlate with the number of CFU grown on plates after 48 h at 30°C.

### Loss of cell membrane integrity.

Propidium iodide was used to evaluate the loss of selective permeability of the membrane after treatments. Cells were stained with 5 μl propidium iodide (50 μg/ml) for 5 min at room temperature. The percentage of positive cells was calculated by observation on a fluorescence microscope.

### Externalization of PS.

Phosphatidylserine (PS) externalization was evaluated by staining protoplasted cells with annexin V-fluorescein isothiocyanate (FITC) (TaKaRa). Protoplasts were generated as previously described (75). Briefly, C. albicans cells were incubated with 0.5 ml 50 mM K_2_HPO_4_, 5 mM EDTA, and 50 mM dithiothreitol (DTT) (adjusted to pH 7.2) at 30°C for 30 min to promote spheroplast formation. After that, 0.5 ml solution containing 50 mM KH_2_PO_4_, 40 mM 2-mercaptoethanol, 0.15 mg/ml Zymolyase 20T, and 20 μl glusulase in 2.4 M sorbitol (pH 7.2) was added and incubated for 30 min at 30°C and 80 rpm. Protoplasts were stained following manufacturer instructions from the ApoAlert annexin V-FITC kit (TaKaRa) in modified annexin binding buffer containing 1.2 M sorbitol. Flow cytometry was used to determine the percentage of cells annexin positive, indicating early and late apoptosis.

### ROS detection.

C. albicans cells from control and treatments were washed thrice in cold PBS and stained with 5 μg/ml dihydrorhodamine 123 (DHR-123; Sigma) for ROS detection 30 min before finishing the experiment (75). The percentage of ROS-positive cells was evaluated with a fluorescence microscope by counting at least 100 cells from at least 3 biological replicates.

### Caspase-like enzymatic activity.

The increase in caspase like enzymatic activity was evaluated by using a staining solution containing (FITC-)VAD-FMK (CaspACE; Promega) at a final concentration of 10 μM/ml (75). Cells were incubated for 20 min at 37°C, washed twice with PBS, and counted in a fluorescence microscope.

### Preparation of soluble protein extracts and peptide digestion for mass spectrometry.

Cells from control and treated samples were harvested and washed thrice in cold PBS. Then cells were resuspended in lysis buffer (50 mM Tris-HCl [pH 7.5], 1 mM EDTA, 150 mM NaCl, 1 mM DTT, 0.5 mM phenylmethylsulfonyl fluoride [PMSF], and 10% of a mix of protease inhibitors (Pierce) and disrupted by adding glass beads (0.4- to 0.6-mm diameter) in a Fast-Prep system (Bio101; Savant) applying 5 cycles of 21 s. Cell extracts were separated from glass beads by centrifugation, and the supernatant was collected and cleared by centrifugation at 13,000 rpm for 15 min at 4°C. Protein concentration was measured by using the Bradford assay, and 50-μg protein extracts were prepared for digestion.

Peptide digestion for SRM and DIA was performed using 50-μg cytoplasmic extracts that were denatured with 8 M urea in 100 mM ammonium bicarbonate and reduced with 5 mM Tris(2-carboxyethyl)phosphine for 30 min at 37°C. After that samples were alkylated with 10 mM iodoacetamide for 45 min in the dark. Finally, samples were digested by trypsin (1/100, wt/wt; Promega) for 16 h at 37°C. Peptides were purified using reverse-phase C_18_ columns. Peptide quantitation was performed using bicinchoninic acid (BCA; Pierce quantitative colorimetric peptide assay) or the Qubit (Thermo Scientific) system.

### DDA and DIA setup.

All global proteomic analyses were performed on a Q Exactive Plus mass spectrometer (Thermo Scientific) connected to an EASY-nLC 1000 ultrahigh-performance liquid chromatography system (Thermo Scientific). Peptides were separated on an EASY-Spray column with a linear gradient of 5 to 30% acetonitrile (ACN) in aqueous 0.1% formic acid (FA) for 60 or 120 min for DDA or DIA, respectively. Settings for DDA and DIA analyses were the same as previously described by Malmström et al. (76). Acquired spectra were analyzed using the search engine X! Tandem (Jackhammer, 2013.06.15) against Assembly 21 (A21-s02-m09-r10) from the Candida Genome Database (CGD [77]) or synthetic retention time peptide sequences with reverse decoys (78). X!Tandem parameters were fixed to 1 missed peptide cleavage and the following modifications: fixed carbamidomethylation of cysteine and the N-terminal variable modifications acetylation, cyclization of S-carbamoylmethylcysteine, and pyroglutamic acid formation of glutamic acid and glutamine. The precursor and fragment mass tolerances were set to 20 ppm and 50 ppm, respectively. Peptide spectrum matches were filtered to a false-discovery rate (FDR) of >1%.

The spectral library was generated on DDA mode from 52 samples from C. albicans upon different conditions (unpublished data deposited at the PRIDE repository, project accession number PXD020195). The assay library was assembled using Fraggle, which interprets and averages MS2 spectra. After, we used Franklin to perform a multilevel FDR calculation, and finally, the assay library was generated with Tramler (79).

The C. albicans spectral library generated was used to extract DIA data from each condition using DIANA algorithm v2.0.0 with FDR correction set to 1% for identification and quantification (80). For protein quantification, the top 3 integrated peptide ion intensities from MS2 spectra were considered (81).

### DIA MS data analysis and GO term enrichment.

For relative quantitation, protein intensities were previously normalized within each sample. Proteins identified in at least 3 biological replicates were considered for quantitative analysis. Statistical analysis by paired *t* test from at least 3 biological replicates was performed. Significant changes in the abundance of proteins between control and treatments were considered when the *P* value was <0.05 and the log_2_ fold change was more than 0.5 or less than −0.5 (paired *t* test from at least 3 biological replicates).

Significant proteins were selected to perform GO term analysis using the GO Term Finder tool from CGD and Genecodis (77, 82). Representative biological processes and cellular component altered after treatments were selected according to *P* value and number of proteins included.

Heatmaps and volcano plots were performed using RStudio (v1.0.143) using ggplot2 and pheatmap.

### SRM design and quantitation.

### (i) Peptide/transition selection and validation.

Targeted proteomic assays were performed according to the method previously reported (83). A set of 32 proteins involved in yeast apoptosis was selected for the targeted proteomic analysis based on previous works published. A total of 63 proteotypic peptides (peptide uniquely associated with the protein of interest) from the selected proteins were designated for SRM relative quantification based on information from PeptideAtlas (84). The same peptides were purchased as synthetic heavy labeled peptides (JPT Peptide Technologies) and used in unpurified form. To confirm peptide identities and select the best three transitions for the SRM method, fragment ion spectra from each peptide included in yeast matrix were acquired by MS2 analysis in a QTRAP 5500 instrument (AB/SCIEX). The data were also used to determine retention times and optimize the method in schedule mode. An adjusted amount of each synthetic peptide was added to a final solution containing all peptides according to their signal intensities. The ratio of synthetic peptides added to the peptide sample was 1:5.

### (ii) SRM mass spectrometer configuration.

Digested samples with synthetic peptides included were injected into an nHPLC (Eksigent nano LC 1D plus) where peptides were concentrated in a trap column (Eksigent nanoLC trap) for 5 min at a flow of 2 μl/min of 2% ACN and 0.1% FA before their separation in a 15-cm analytical column (Eksigent nanoLC column 2C18-CL). Peptide elution was achieved by a 5 to 35% ACN gradient for 30 min at 300 nl/min. Both trap and analytical columns were heated at 50°C to get more constant retention times between samples. At least three biological replicates of each condition were analyzed, with at least 2 technical replicates of each one. All analyses were performed in an AB Sciex QTRAP 5500 mass spectrometer.

### (iii) SRM data analysis.

The raw files containing SRM data were analyzed with Skyline software (v 4.2.0.19009), and the peak selection in the chromatograms of each peptide was manually curated (85). Transitions showing some interference in peak area were excluded. The intensity area of each peak was automatically calculated by the software considering the value as the endogenous/synthetic precursor ratio. The statistical tool implemented in Skyline software reported the significant difference of endogenous peptide between samples (*t* test < 0.05).

### Determination of enzymatic activity of the proteasome.

For the measurement of proteasome activity, a fluorometric assay kit (proteasome 20S activity assay kit) that measures the chymotrypsin-like protease activity was used following manufacturer instructions (Sigma-Aldrich). One-hundred-microgram protein extracts were incubated with LLVY-R110 substrate provided by the kit for 2 h at 30°C in the dark. The green fluorescent signal generated by the cleavage of LLVY-R110 by proteasome was measured with BMG FLUOstar Galaxy equipment (λ_ex_ = 480 to 500 nm/λ_em_ = 520 to 530 nm). Data from three technical replicates and three biological replicates measured as fluorescence arbitrary units were used for paired *t* test analysis (*P* value < 0.05).

### Western blotting.

Western blotting was carried out as described in Text S1. Ubiquitin epitopes were detected using an anti-ubiquitin antibody (MAB1510-I anti-ubiquitin clone Ubi-1, diluted 1:500; Millipore). Membranes were stained with 15 ml Pierce reversible stain (Pierce TM 24580) as a loading control.

10.1128/mSystems.00946-21.1 TEXT S1 Preparation of soluble protein extracts and Western blotting. Download Text S1, PDF file, 0.07 MB. Copyright © 2021 Amador-García et al. 2021 Amador-García et al. https://creativecommons.org/licenses/by/4.0/ This content is distributed under the terms of the Creative Commons Attribution 4.0 International license.

### Data availability.

The data set from this paper has been deposited in the ProteomeXchange Consortium via the PRIDE partner repository with the data set identifier PXD020283.

## References

[1] Vincent JL, Rello J, Marshall J, Silva E, Anzueto A, Martin CD, Moreno R, Lipman J, Gomersall C, Sakr Y, Reinhart K, EPIC II Group of Investigators. 2009. International study of the prevalence and outcomes of infection in intensive care units. JAMA 302:2323–2329. doi:10.1001/jama.2009.1754. 19952319

[2] Kullberg BJ, Arendrup MC. 2015. Invasive candidiasis. N Engl J Med 373:1445–1456. doi:10.1056/NEJMra1315399. 26444731

[3] Mayer FL, Wilson D, Hube B. 2013. *Candida albicans* pathogenicity mechanisms. Virulence 4:119–128. doi:10.4161/viru.22913. 23302789 PMC3654610

[4] Dantas A.dS, Day A, Ikeh M, Kos I, Achan B, Quinn J. 2015. Oxidative stress responses in the human fungal pathogen Candida albicans. Biomolecules 5:142–165. doi:10.3390/biom5010142. 25723552 PMC4384116

[5] Mortensen PB, Clausen MR. 1996. Short-chain fatty acids in the human colon: relation to gastrointestinal health and disease. Scand J Gastroenterol Suppl 216:132–148. doi:10.3109/00365529609094568. 8726286

[6] Yeaman MR, Buttner S, Thevissen K. 2018. Regulated cell death as a therapeutic target for novel antifungal peptides and biologics. Oxid Med Cell Longev 2018:5473817. doi:10.1155/2018/5473817. 29854086 PMC5944218

[7] Kulkarni M, Stolp ZD, Hardwick JM. 2019. Targeting intrinsic cell death pathways to control fungal pathogens. Biochem Pharmacol 162:71–78. doi:10.1016/j.bcp.2019.01.012. 30660496 PMC6430978

[8] Phillips AJ, Sudbery I, Ramsdale M. 2003. Apoptosis induced by environmental stresses and amphotericin B in *Candida albicans*. Proc Natl Acad Sci USA 100:14327–14332. doi:10.1073/pnas.2332326100. 14623979 PMC283591

[9] Dai BD, Cao YY, Huang S, Xu YG, Gao PH, Wang Y, Jiang YY. 2009. Baicalein induces programmed cell death in Candida albicans. J Microbiol Biotechnol 19:803–809. 19734718

[10] Shirtliff ME, Krom BP, Meijering RA, Peters BM, Zhu J, Scheper MA, Harris ML, Jabra-Rizk MA. 2009. Farnesol-induced apoptosis in *Candida albicans*. Antimicrob Agents Chemother 53:2392–2401. doi:10.1128/AAC.01551-08. 19364863 PMC2687256

[11] Hao B, Cheng S, Clancy CJ, Nguyen MH. 2013. Caspofungin kills *Candida albicans* by causing both cellular apoptosis and necrosis. Antimicrob Agents Chemother 57:326–332. doi:10.1128/AAC.01366-12. 23114781 PMC3535936

[12] Lee W, Lee Dong G. 2018. A novel mechanism of fluconazole: fungicidal activity through dose-dependent apoptotic responses in *Candida albicans*. Microbiology (Reading) 164:194–204. doi:10.1099/mic.0.000589. 29393017

[13] Thakre A, Zore G, Kodgire S, Kazi R, Mulange S, Patil R, Shelar A, Santhakumari B, Kulkarni M, Kharat K, Karuppayil SM. 2018. Limonene inhibits Candida albicans growth by inducing apoptosis. Med Mycol 56:565–578. doi:10.1093/mmy/myx074. 29420815

[14] Léger T, Ounissi M, Lelandais G. 2015. The metacaspase (Mca1p) has a dual role in Farnesol-induced apoptosis in *Candida albicans*. Mol Cell Proteomics 14:93–108. doi:10.1074/mcp.M114.041210. 25348831 PMC4288266

[15] Carmona-Gutierrez D, Eisenberg T, Buttner S, Meisinger C, Kroemer G, Madeo F. 2010. Apoptosis in yeast: triggers, pathways, subroutines. Cell Death Differ 17:763–773. doi:10.1038/cdd.2009.219. 20075938

[16] Phillips AJ, Crowe JD, Ramsdale M. 2006. Ras pathway signaling accelerates programmed cell death in the pathogenic fungus *Candida albicans*. Proc Natl Acad Sci USA 103:726–731. doi:10.1073/pnas.0506405103. 16407097 PMC1334641

[17] Carmona-Gutierrez D, Bauer MA, Zimmermann A, Aguilera A, Austriaco N, Ayscough K, Balzan R, Bar-Nun S, Barrientos A, Belenky P, Blondel M, Braun RJ, Breitenbach M, Burhans WC, Büttner S, Cavalieri D, Chang M, Cooper KF, Côrte-Real M, Costa V, Cullin C, Dawes I, Dengjel J, Dickman MB, Eisenberg T, Fahrenkrog B, Fasel N, Fröhlich K-U, Gargouri A, Giannattasio S, Goffrini P, Gourlay CW, Grant CM, Greenwood MT, Guaragnella N, Heger T, Heinisch J, Herker E, Herrmann JM, Hofer S, Jiménez-Ruiz A, Jungwirth H, Kainz K, Kontoyiannis DP, Ludovico P, Manon S, Martegani E, Mazzoni C, Megeney LA, Meisinger C, et al. 2018. Guidelines and recommendations on yeast cell death nomenclature. Microb Cell 5:4–31. doi:10.15698/mic2018.01.607. 29354647 PMC5772036

[18] Madeo F, Frohlich E, Frohlich KU. 1997. A yeast mutant showing diagnostic markers of early and late apoptosis. J Cell Biol 139:729–734. doi:10.1083/jcb.139.3.729. 9348289 PMC2141703

[19] Madeo F, Frohlich E, Ligr M, Grey M, Sigrist SJ, Wolf DH, Frohlich KU. 1999. Oxygen stress: a regulator of apoptosis in yeast. J Cell Biol 145:757–767. doi:10.1083/jcb.145.4.757. 10330404 PMC2133192

[20] Gillet LC, Navarro P, Tate S, Rost H, Selevsek N, Reiter L, Bonner R, Aebersold R. 2012. Targeted data extraction of the MS/MS spectra generated by data-independent acquisition: a new concept for consistent and accurate proteome analysis. Mol Cell Proteomics 11:O111.016717. doi:10.1074/mcp.O111.016717. PMC3433915 22261725

[21] Gallien S, Duriez E, Domon B. 2011. Selected reaction monitoring applied to proteomics. J Mass Spectrom 46:298–312. doi:10.1002/jms.1895. 21394846

[22] Yang C, Gong W, Lu J, Zhu X, Qi Q. 2010. Antifungal drug susceptibility of oral Candida albicans isolates may be associated with apoptotic responses to amphotericin B. J Oral Pathol Med 39:182–187. doi:10.1111/j.1600-0714.2009.00811.x. 19656268

[23] Komalapriya C, Kaloriti D, Tillmann AT, Yin Z, Herrero-de-Dios C, Jacobsen MD, Belmonte RC, Cameron G, Haynes K, Grebogi C, de Moura AP, Gow NA, Thiel M, Quinn J, Brown AJ, Romano MC. 2015. Integrative model of oxidative stress adaptation in the fungal pathogen *Candida albicans*. PLoS One 10:e0137750. doi:10.1371/journal.pone.0137750. 26368573 PMC4569071

[24] Fernandez MP, Biscoglio MJ, Passeron S. 2000. Purification and characterization of Candida albicans 20S proteasome: identification of four proteasomal subunits. Arch Biochem Biophys 375:211–219. doi:10.1006/abbi.1999.1591. 10700377

[25] Berger TM, Herrmann JM, Vielhauer V, Luckow B, Detlef K. 2000. The apoptosis mediator mDAP-3 is a novel member of a conserved family of mitochondrial proteins. J Cell Sci 113 (Part 20):3603–3612. doi:10.1242/jcs.113.20.3603. 11017876

[26] Buttner S, Ruli D, Vogtle FN, Galluzzi L, Moitzi B, Eisenberg T, Kepp O, Habernig L, Carmona-Gutierrez D, Rockenfeller P, Laun P, Breitenbach M, Khoury C, Frohlich KU, Rechberger G, Meisinger C, Kroemer G, Madeo F. 2011. A yeast BH3-only protein mediates the mitochondrial pathway of apoptosis. EMBO J 30:2779–2792. doi:10.1038/emboj.2011.197. 21673659 PMC3160254

[27] Cebulski J, Malouin J, Pinches N, Cascio V, Austriaco N. 2011. Yeast Bax inhibitor, Bxi1p, is an ER-localized protein that links the unfolded protein response and programmed cell death in *Saccharomyces cerevisiae*. PLoS One 6:e20882. doi:10.1371/journal.pone.0020882. 21673967 PMC3108976

[28] Fannjiang Y, Cheng WC, Lee SJ, Qi B, Pevsner J, McCaffery JM, Hill RB, Basanez G, Hardwick JM. 2004. Mitochondrial fission proteins regulate programmed cell death in yeast. Genes Dev 18:2785–2797. doi:10.1101/gad.1247904. 15520274 PMC528898

[29] Fernández-Arenas E, Cabezón V, Bermejo C, Arroyo J, Nombela C, Diez-Orejas R, Gil C. 2007. Integrated proteomics and genomics strategies bring new insight into *Candida albicans* response upon macrophage interaction. Mol Cell Proteomics 6:460–478. doi:10.1074/mcp.M600210-MCP200. 17164403

[30] Heiden Matthew GV, Cho JS, VanderWeele DJ, Brace JL, Harris MH, Bauer DE, Prange B, Kron SJ, Thompson CB, Rudin CM. 2002. Bcl-xL complements *Saccharomyces cerevisiae* genes that facilitate the switch from glycolytic to oxidative metabolism. J Biol Chem 277:44870–44876. doi:10.1074/jbc.M2048882000. 12244097

[31] Hill SM, Nystrom T. 2015. The dual role of a yeast metacaspase: what doesn’t kill you makes you stronger. Bioessays 37:525–531. doi:10.1002/bies.201400208. 25677381 PMC5053244

[32] Hromatka BS, Noble SM, Johnson AD. 2005. Transcriptional response of *Candida albicans* to nitric oxide and the role of the YHB1 gene in nitrosative stress and virulence. Mol Biol Cell 16:4814–4826. doi:10.1091/mbc.e05-05-0435. 16030247 PMC1237085

[33] Kazemzadeh L, Cvijovic M, Petranovic D. 2012. Boolean model of yeast apoptosis as a tool to study yeast and human apoptotic regulations. Front Physiol 3:446. doi:10.3389/fphys.2012.00446. 23233838 PMC3518040

[34] Kitahara N, Morisaka H, Aoki W, Takeda Y, Shibasaki S, Kuroda K, Ueda M. 2015. Description of the interaction between *Candida albicans* and macrophages by mixed and quantitative proteome analysis without isolation. AMB Express 5:127. doi:10.1186/s13568-015-0127-2. 26179440 PMC4503712

[35] Liang Q, Li W, Zhou B. 2008. Caspase-independent apoptosis in yeast. Biochim Biophys Acta 1783:1311–1319. doi:10.1016/j.bbamcr.2008.02.018. 18358844

[36] Ligr M, Velten I, Fröhlich E, Madeo F, Ledig M, Fröhlich KU, Wolf DH, Hilt W. 2001. The proteasomal substrate Stm1 participates in apoptosis-like cell death in yeast. Mol Biol Cell 12:2422–2432. doi:10.1091/mbc.12.8.2422. 11514626 PMC58604

[37] Ludovico P, Rodrigues F, Almeida A, Silva MT, Barrientos A, Côrte-Real M. 2002. Cytochrome c release and mitochondria involvement in programmed cell death induced by acetic acid in *Saccharomyces cerevisiae*. Mol Biol Cell 13:2598–2606. doi:10.1091/mbc.e01-12-0161. 12181332 PMC117928

[38] Madeo F, Carmona-Gutierrez D, Ring J, Büttner S, Eisenberg T, Kroemer G. 2009. Caspase-dependent and caspase-independent cell death pathways in yeast. Biochem Biophys Res Commun 382:227–231. doi:10.1016/j.bbrc.2009.02.117. 19250922

[39] Madeo F, Herker E, Maldener C, Wissing S, Lachelt S, Herlan M, Fehr M, Lauber K, Sigrist SJ, Wesselborg S, Frohlich KU. 2002. A caspase-related protease regulates apoptosis in yeast. Mol Cell 9:911–917. doi:10.1016/s1097-2765(02)00501-4. 11983181

[40] Manon S, Chaudhuri B, Guerin M. 1997. Release of cytochrome c and decrease of cytochrome c oxidase in Bax-expressing yeast cells, and prevention of these effects by coexpression of Bcl-xL. FEBS Lett 415:29–32. doi:10.1016/s0014-5793(97)01087-9. 9326363

[41] Odat O, Matta S, Khalil H, Kampranis SC, Pfau R, Tsichlis PN, Makris AM. 2007. Old yellow enzymes, highly homologous FMN oxidoreductases with modulating roles in oxidative stress and programmed cell death in yeast. J Biol Chem 282:36010–36023. doi:10.1074/jbc.M704058200. 17897954

[42] Pereira C, Silva RD, Saraiva L, Johansson B, Sousa MJ, Côrte-Real M. 2008. Mitochondria-dependent apoptosis in yeast. Biochim Biophys Acta 1783:1286–1302. doi:10.1016/j.bbamcr.2008.03.010. 18406358

[43] Pozniakovsky AI, Knorre DA, Markova OV, Hyman AA, Skulachev VP, Severin FF. 2005. Role of mitochondria in the pheromone- and amiodarone-induced programmed death of yeast. J Cell Biol 168:257–269. doi:10.1083/jcb.200408145. 15657396 PMC2171581

[44] Qiu J, Yoon JH, Shen B. 2005. Search for apoptotic nucleases in yeast: role of Tat-D nuclease in apoptotic DNA degradation. J Biol Chem 280:15370–15379. doi:10.1074/jbc.M413547200. 15657035

[45] Rinnerthaler M, Jarolim S, Heeren G, Palle E, Perju S, Klinger H, Bogengruber E, Madeo F, Braun RJ, Breitenbach-Koller L, Breitenbach M, Laun P. 2006. MMI1 (YKL056c, TMA19), the yeast orthologue of the translationally controlled tumor protein (TCTP) has apoptotic functions and interacts with both microtubules and mitochondria. Biochim Biophys Acta 1757:631–638. doi:10.1016/j.bbabio.2006.05.022. 16806052

[46] Silva A, Almeida B, Sampaio-Marques B, Reis MI, Ohlmeier S, Rodrigues F, Vale A, Ludovico P. 2011. Glyceraldehyde-3-phosphate dehydrogenase (GAPDH) is a specific substrate of yeast metacaspase. Biochim Biophys Acta 1813:2044–2049. doi:10.1016/j.bbamcr.2011.09.010. 21982825

[47] Szallies A, Kubata BK, Duszenko M. 2002. A metacaspase of *Trypanosoma brucei* causes loss of respiration competence and clonal death in the yeast *Saccharomyces cerevisiae*. FEBS Lett 517:144–150. doi:10.1016/s0014-5793(02)02608-x. 12062425

[48] Thompson DM, Parker R. 2009. The RNase Rny1p cleaves tRNAs and promotes cell death during oxidative stress in *Saccharomyces cerevisiae*. J Cell Biol 185:43–50. doi:10.1083/jcb.200811119. 19332891 PMC2700514

[49] Vachova L, Palkova Z. 2005. Physiological regulation of yeast cell death in multicellular colonies is triggered by ammonia. J Cell Biol 169:711–717. doi:10.1083/jcb.200410064. 15939758 PMC2171614

[50] Vendrell A, Martínez-Pastor M, González-Novo A, Pascual-Ahuir A, Sinclair DA, Proft M, Posas F. 2011. Sir2 histone deacetylase prevents programmed cell death caused by sustained activation of the Hog1 stress-activated protein kinase. EMBO Rep 12:1062–1068. doi:10.1038/embor.2011.154. 21836634 PMC3185340

[51] Walter D, Wissing S, Madeo F, Fahrenkrog B. 2006. The inhibitor-of-apoptosis protein Bir1p protects against apoptosis in *S. cerevisiae* and is a substrate for the yeast homologue of Omi/HtrA2. J Cell Sci 119:1843–1851. doi:10.1242/jcs.02902. 16608876

[52] Wang Y, Cao YY, Jia XM, Cao YB, Gao PH, Fu XP, Ying K, Chen WS, Jiang YY. 2006. Cap1p is involved in multiple pathways of oxidative stress response in *Candida albicans*. Free Radic Biol Med 40:1201–1209. doi:10.1016/j.freeradbiomed.2005.11.019. 16545688

[53] Wissing S, Ludovico P, Herker E, Buttner S, Engelhardt SM, Decker T, Link A, Proksch A, Rodrigues F, Corte-Real M, Frohlich KU, Manns J, Cande C, Sigrist SJ, Kroemer G, Madeo F. 2004. An AIF orthologue regulates apoptosis in yeast. J Cell Biol 166:969–974. doi:10.1083/jcb.200404138. 15381687 PMC2172025

[54] Zaid H, Abu-Hamad S, Israelson A, Nathan I, Shoshan-Barmatz V. 2005. The voltage-dependent anion channel-1 modulates apoptotic cell death. Cell Death Differ 12:751–760. doi:10.1038/sj.cdd.4401599. 15818409

[55] Kusch H, Engelmann S, Albrecht D, Morschhauser J, Hecker M. 2007. Proteomic analysis of the oxidative stress response in Candida albicans. Proteomics 7:686–697. doi:10.1002/pmic.200600575. 17285563

[56] Yin Z, Stead D, Walker J, Selway L, Smith DA, Brown AJ, Quinn J. 2009. A proteomic analysis of the salt, cadmium and peroxide stress responses in Candida albicans and the role of the Hog1 stress-activated MAPK in regulating the stress-induced proteome. Proteomics 9:4686–4703. doi:10.1002/pmic.200800958. 19824012

[57] Costa V, Quintanilha A, Moradas-Ferreira P. 2007. Protein oxidation, repair mechanisms and proteolysis in *Saccharomyces cerevisiae*. IUBMB Life 59:293–298. doi:10.1080/15216540701225958. 17505968

[58] Orzaez D, de Jong AJ, Woltering EJ. 2001. A tomato homologue of the human protein PIRIN is induced during programmed cell death. Plant Mol Biol 46:459–468. doi:10.1023/a:1010618515051. 11485202

[59] Gelbman BD, Heguy A, O’Connor TP, Zabner J, Crystal RG. 2007. Upregulation of pirin expression by chronic cigarette smoking is associated with bronchial epithelial cell apoptosis. Respir Res 8:10. doi:10.1186/1465-9921-8-10. 17288615 PMC1805431

[60] Tala A, Damiano F, Gallo G, Pinatel E, Calcagnile M, Testini M, Fico D, Rizzo D, Sutera A, Renzone G, Scaloni A, De Bellis G, Siculella L, De Benedetto GE, Puglia AM, Peano C, Alifano P. 2018. Pirin: a novel redox-sensitive modulator of primary and secondary metabolism in *Streptomyces*. Metab Eng 48:254–268. doi:10.1016/j.ymben.2018.06.008. 29944936

[61] Cottier F, Tan AS, Chen J, Lum J, Zolezzi F, Poidinger M, Pavelka N. 2015. The transcriptional stress response of *Candida albicans* to weak organic acids. G3 (Bethesda) 5:497–505. doi:10.1534/g3.114.015941. 25636313 PMC4390566

[62] Cottier F, Tan ASM, Yurieva M, Liao W, Lum J, Poidinger M, Zolezzi F, Pavelka N. 2017. The transcriptional response of *Candida albicans* to weak organic acids, carbon source, and MIG1 inactivation unveils a role for HGT16 in mediating the fungistatic effect of acetic acid. G3 (Bethesda) 7:3597–3604. doi:10.1534/g3.117.300238. 28877970 PMC5677169

[63] Almeida B, Ohlmeier S, Almeida AJ, Madeo F, Leao C, Rodrigues F, Ludovico P. 2009. Yeast protein expression profile during acetic acid-induced apoptosis indicates causal involvement of the TOR pathway. Proteomics 9:720–732. doi:10.1002/pmic.200700816. 19137548

[64] Dong Y, Hu J, Fan L, Chen Q. 2017. RNA-Seq-based transcriptomic and metabolomic analysis reveal stress responses and programmed cell death induced by acetic acid in *Saccharomyces cerevisiae*. Sci Rep 7:42659. doi:10.1038/srep42659. 28209995 PMC5314350

[65] Sousa M, Duarte AM, Fernandes TR, Chaves SR, Pacheco A, Leao C, Corte-Real M, Sousa MJ. 2013. Genome-wide identification of genes involved in the positive and negative regulation of acetic acid-induced programmed cell death in *Saccharomyces cerevisiae*. BMC Genomics 14:838. doi:10.1186/1471-2164-14-838. 24286259 PMC4046756

[66] Longo V, Ždralević M, Guaragnella N, Giannattasio S, Zolla L, Timperio AM. 2015. Proteome and metabolome profiling of wild-type and YCA1-knock-out yeast cells during acetic acid-induced programmed cell death. J Proteomics 128:173–188. doi:10.1016/j.jprot.2015.08.003. 26269384

[67] Sung MK, Reitsma JM, Sweredoski MJ, Hess S, Deshaies RJ. 2016. Ribosomal proteins produced in excess are degraded by the ubiquitin-proteasome system. Mol Biol Cell 27:2642–2652. doi:10.1091/mbc.E16-05-0290. 27385339 PMC5007085

[68] Tomecki R, Sikorski PJ, Zakrzewska-Placzek M. 2017. Comparison of preribosomal RNA processing pathways in yeast, plant and human cells—focus on coordinated action of endo- and exoribonucleases. FEBS Lett 591:1801–1850. doi:10.1002/1873-3468.12682. 28524231

[69] Gupta I, Singh K, Varshney NK, Khan S. 2018. Delineating crosstalk mechanisms of the ubiquitin proteasome system that regulate apoptosis. Front Cell Dev Biol 6:11. doi:10.3389/fcell.2018.00011. 29479529 PMC5811474

[70] Lee DH, Goldberg AL. 1998. Proteasome inhibitors: valuable new tools for cell biologists. Trends Cell Biol 8:397–403. doi:10.1016/s0962-8924(98)01346-4. 9789328

[71] Kaneko Y, Fukazawa H, Ohno H, Miyazaki Y. 2013. Combinatory effect of fluconazole and FDA-approved drugs against Candida albicans. J Infect Chemother 19:1141–1145. doi:10.1007/s10156-013-0639-0. 23807392

[72] Worboys JD, Sinclair J, Yuan Y, Jorgensen C. 2014. Systematic evaluation of quantotypic peptides for targeted analysis of the human kinome. Nat Methods 11:1041–1044. doi:10.1038/nmeth.3072. 25152083 PMC4180722

[73] Leiter E, Csernoch L, Pocsi I. 2018. Programmed cell death in human pathogenic fungi—a possible therapeutic target. Expert Opin Ther Targets 22:1039–1048. doi:10.1080/14728222.2018.1541087. 30360667

[74] Noble SM, French S, Kohn LA, Chen V, Johnson AD. 2010. Systematic screens of a *Candida albicans* homozygous deletion library decouple morphogenetic switching and pathogenicity. Nat Genet 42:590–598. doi:10.1038/ng.605. 20543849 PMC2893244

[75] Cabezón V, Vialás V, Gil-Bona A, Reales-Calderón JA, Martínez-Gomariz M, Gutiérrez-Blázquez D, Monteoliva L, Molero G, Ramsdale M, Gil C. 2016. Apoptosis of *Candida albicans* during the interaction with murine macrophages: proteomics and cell-death marker monitoring. J Proteome Res 15:1418–1434. doi:10.1021/acs.jproteome.5b00913. 27048922

[76] Malmström E, Kilsgard O, Hauri S, Smeds E, Herwald H, Malmström L, Malmström J. 2016. Large-scale inference of protein tissue origin in gram-positive sepsis plasma using quantitative targeted proteomics. Nat Commun 7:10261. doi:10.1038/ncomms10261. 26732734 PMC4729823

[77] Skrzypek MS, Binkley J, Binkley G, Miyasato SR, Simison M, Sherlock G. 2017. The Candida Genome Database (CGD): incorporation of Assembly 22, systematic identifiers and visualization of high throughput sequencing data. Nucleic Acids Res 45:D592–D596. doi:10.1093/nar/gkw924. 27738138 PMC5210628

[78] Escher C, Reiter L, MacLean B, Ossola R, Herzog F, Chilton J, MacCoss MJ, Rinner O. 2012. Using iRT, a normalized retention time for more targeted measurement of peptides. Proteomics 12:1111–1121. doi:10.1002/pmic.201100463. 22577012 PMC3918884

[79] Teleman J, Hauri S, Malmstrom J. 2017. Improvements in mass spectrometry assay library generation for targeted proteomics. J Proteome Res 16:2384–2392. doi:10.1021/acs.jproteome.6b00928. 28516777

[80] Teleman J, Rost HL, Rosenberger G, Schmitt U, Malmstrom L, Malmstrom J, Levander F. 2015. DIANA—algorithmic improvements for analysis of data-independent acquisition MS data. Bioinformatics 31:555–562. doi:10.1093/bioinformatics/btu686. 25348213

[81] Ahrne E, Molzahn L, Glatter T, Schmidt A. 2013. Critical assessment of proteome-wide label-free absolute abundance estimation strategies. Proteomics 13:2567–2578. doi:10.1002/pmic.201300135. 23794183

[82] Nogales-Cadenas R, Carmona-Saez P, Vazquez M, Vicente C, Yang X, Tirado F, Carazo JM, Pascual-Montano A. 2009. GeneCodis: interpreting gene lists through enrichment analysis and integration of diverse biological information. Nucleic Acids Res 37:W317–W322. doi:10.1093/nar/gkp416. 19465387 PMC2703901

[83] Picotti P, Rinner O, Stallmach R, Dautel F, Farrah T, Domon B, Wenschuh H, Aebersold R. 2010. High-throughput generation of selected reaction-monitoring assays for proteins and proteomes. Nat Methods 7:43–46. doi:10.1038/nmeth.1408. 19966807

[84] Vialas V, Sun Z, Reales-Calderón JA, Hernáez ML, Casas V, Carrascal M, Abián J, Monteoliva L, Deutsch EW, Moritz RL, Gil C. 2016. A comprehensive *Candida albicans* PeptideAtlas build enables deep proteome coverage. J Proteomics 131:122–130. doi:10.1016/j.jprot.2015.10.019. 26493587 PMC4670622

[85] Bereman MS, MacLean B, Tomazela DM, Liebler DC, MacCoss MJ. 2012. The development of selected reaction monitoring methods for targeted proteomics via empirical refinement. Proteomics 12:1134–1141. doi:10.1002/pmic.201200042. 22577014 PMC3643124

