# Supplementary material for: Extending the Proteomic Characterization of Candida albicans Exposed to Stress and Apoptotic Inducers through Data-Independent Acquisition Mass Spectrometry
Source: mSystems. 2021 Oct 5;6(5):e00946-21. doi: 10.1128/mSystems.00946-21 (PMC8547427; doi:10.1128/mSystems.00946-21)
Supplement: FIG S1 [file msystems.00946-21-sf001.pdf]

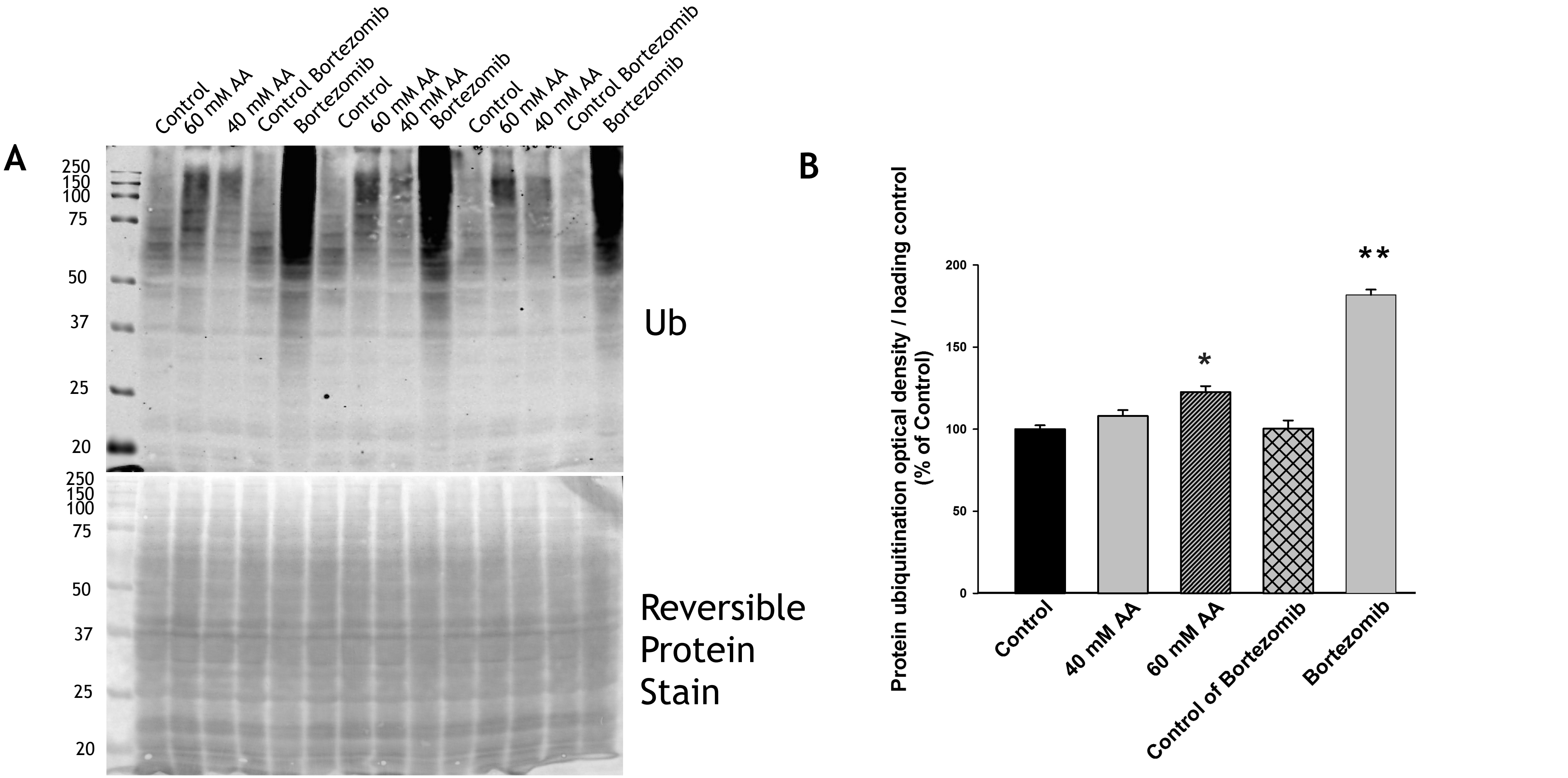

Fig 1. A. Protein ubiquitination in response to the treatment with different doses (40 and 60 mM) of acetic acid (AA). The inhibition of the proteasome by Bortezomib (proteasome inhibitor) is used as positive control. B. Western blot analysis of changes induced by treatment with AA in the expression of protein ubiquitination in controls, 40 mM AA and 60 mM AA groups. Data are means ± SEM. **p<0.001 relative to the corresponding control group, *p<0.05 relative to the control group . (One-way ANOVA and Bonferroni post-hoc test).
