# Supplementary material for: Extending the Proteomic Characterization of Candida albicans Exposed to Stress and Apoptotic Inducers through Data-Independent Acquisition Mass Spectrometry
Source: mSystems. 2021 Oct 5;6(5):e00946-21. doi: 10.1128/mSystems.00946-21 (PMC8547427; doi:10.1128/mSystems.00946-21)
Supplement: TEXT S1 [file msystems.00946-21-s0001.pdf]

Supplemental methods

S1. Preparation of soluble protein extracts and Western blotting

C. albicans strain SC5314 was maintained in yeast extract-peptone-dextrose (YPD) medium at 30°C. Pre-inoculum was adjusted to an optical density between 0.8 and 1, and then incubated for 200 min in the absence or presence of 40 mM or 60 mM acetic acid (AA). In addition, it was adjusted to an optical density between 0.02 and 0.1, and then incubated for 7 h in the absence or presence of 260 μM bortezomib (inhibitor of proteasome) (CAS 179324-69-7, Merck) at 30°C with shaking. Cells from control and treated samples were harvested and washed thrice in cold PBS. These were then resuspended in lysis buffer (50 mM Tris-HCl pH 7.5, 1 mM EDTA, 150 mM NaCl, 1 mM DTT, 0.5 mM PMSF, and 10 % of a mix of protease inhibitors (Pierce TM), and disrupted by adding glass beads (0.4-0.6 mm diameter) in a Fast-Prep system (Bio101, Savant) applying 5 cycles of 21 sec. Cell extracts were separated from glass beads by centrifugation, and the supernatant was collected and cleared by centrifugation at 13000 rpm for 15 min at 4ºC. Protein concentration was measured with the Bradford assay (Protein Assay Dye Reagent Concentrate, BioRad). Samples were heated at 95°C for 5 minutes and debris was pelleted by spinning for 1 minute at 14000 rpm. Forty micrograms of protein per sample was separated by SDS-PAGE using 15-well 10% gels. Separated proteins were electrotransferred to nitrocellulose membrane (GE Healthcare) for 60 minutes at 300 mA. Membranes were then washed with distilled water and stained with 15 mL Pierce Reversible Stain (Pierce TM 24580) for 5 minutes. Membranes were washed 2-3 times with distilled water and imaged. Blots were blocked with 10% skim milk in PBS with 0.1% Tween 20 (PBS-T) for 90 min. Ubiquitin epitopes were detected using an anti-ubiquitin antibody (1:500, clone Ubi-1, MAB1510-I, Millipore) in 5% skim milk in PBS-T overnight at 4°C. Blots were washed with PBS-T and incubated with Alexa Fluor Plus 800 secondary antibody diluted in 5% skim milk in PBS-T for 1 h. Signals were detected using Odyssey Fc Imaging System as indicated by the manufacturer’s instructions (LI-COR Biosciences).
